# The Spectrum of Asynchronous Dynamics in Spiking Networks as a Model for the Diversity of Non-rhythmic Waking States in the Neocortex

**DOI:** 10.1016/j.celrep.2019.03.102

**Published:** 2019-04-23

**Authors:** Yann Zerlaut, Stefano Zucca, Stefano Panzeri, Tommaso Fellin

**Affiliations:** 1 Neural Coding Laboratory, Istituto Italiano di Tecnologia, Genova, Italy; 2 Neural Computation Laboratory, Center for Neuroscience and Cognitive Systems @UniTn, Istituto Italiano di Tecnologia, Rovereto, Italy; 3 Optical Approaches to Brain Function Laboratory, Istituto Italiano di Tecnologia, Genova, Italy

**Keywords:** waking states, neocortical dynamics, spiking network models, state-dependent computation, somatosensory cortex

## Abstract

The awake cortex exhibits diverse non-rhythmic network states. However, how these states emerge and how each state impacts network function is unclear. Here, we demonstrate that model networks of spiking neurons with moderate recurrent interactions display a spectrum of non-rhythmic asynchronous dynamics based on the level of afferent excitation, from afferent input-dominated (AD) regimes, characterized by unbalanced synaptic currents and sparse firing, to recurrent input-dominated (RD) regimes, characterized by balanced synaptic currents and dense firing. The model predicted regime-specific relationships between different neural biophysical properties, which were all experimentally validated in the somatosensory cortex (S1) of awake mice. Moreover, AD regimes more precisely encoded spatiotemporal patterns of presynaptic activity, while RD regimes better encoded the strength of afferent inputs. These results provide a theoretical foundation for how recurrent neocortical circuits generate non-rhythmic waking states and how these different states modulate the processing of incoming information.

## Highlights

• Awake primary somatosensory cortex exhibits diverse non-rhythmic asynchronous dynamics
• A recurrent network model explains how such dynamics may originate
• The model explains well the dynamics of the waking somatosensory cortex
• The model predicts how non-rhythmic waking dynamics modulates information processing

## Graphical Abstract

**Figure:**
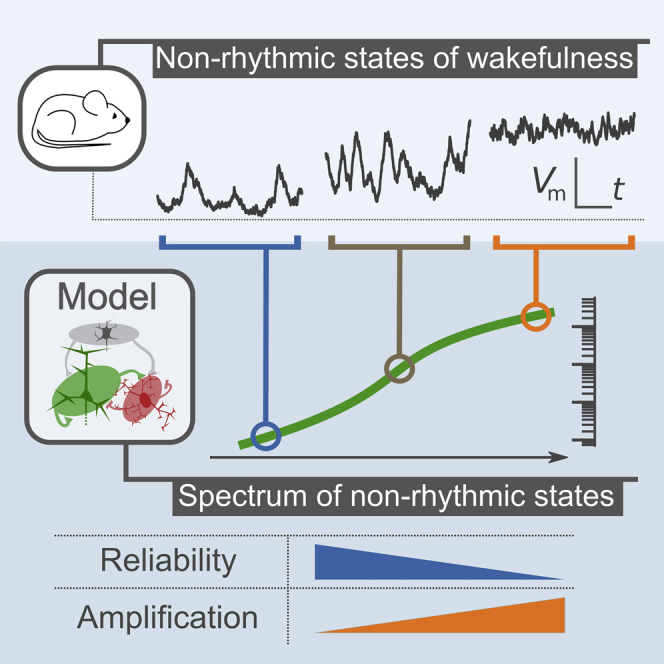

## Teaser

Zerlaut et al. develop a recurrent neural network model that explains several aspects of the asynchronous non-rhythmic dynamics observed in the somatosensory cortex of waking mice. This model predicts how networks with moderate recurrent interactions can profoundly and dynamically modulate the cell membrane potential, the network firing rate, and information coding properties.

## Introduction

Cortical circuits display spontaneous asynchronous dynamics with low pairwise spiking synchrony (Ecker et al., 2010, Renart et al., 2010). Theoretical description of these regimes is based on balanced synaptic activity emerging from recurrent networks (Amit and Brunel, 1997, Destexhe and Contreras, 2006, Hennequin et al., 2017, Kumar et al., 2008, Litwin-Kumar and Doiron, 2012, Parga, 2013, Renart et al., 2010, Tsodyks and Sejnowski, 1995, Vogels et al., 2005, van Vreeswijk and Sompolinsky, 1996). In this setting, excitatory and inhibitory currents cancel each other and generate Gaussian fluctuations in the membrane potential (*V*_*m*_) with a mean close to the spiking threshold (van Vreeswijk and Sompolinsky, 1996). While early recordings in cats supported this view (Steriade et al., 2001), recent experiments in awake rodents suggest a more complex picture (Busse et al., 2017, McGinley et al., 2015a, Nakajima and Halassa, 2017): spontaneous cortical dynamics exhibits diverse asynchronous states characterized by different mean *V*_*m*_ (McGinley et al., 2015b, Polack et al., 2013, Reimer et al., 2014) and firing activity (Vinck et al., 2015).

These observations raise fundamental questions. Is recurrently balanced dynamics a valid model for all the different asynchronous states? If not, do asynchronous dynamics exist beyond the balanced setting? Can we develop a computational model that reveals the mechanisms generating these asynchronous states, that precisely describes the *V*_*m*_ dynamics observed during wakefulness, and that allows us to understand the specific computational advantages of each state?

To address these questions, we explored the dynamics emerging in models of recurrently connected networks of excitatory and inhibitory spiking units. We found that, for moderate recurrent interactions, spiking network models displayed a spectrum of asynchronous states exhibiting spiking activity spanning over orders of magnitudes, with profound differences across states in the relative contributions of the afferent and recurrent components to network dynamics. The model predicted a number of relationships among different electrophysiologically measurable features that were all experimentally confirmed by electrophysiological recordings of neural activity in the mouse somatosensory cortex (S1). Finally, we demonstrated that different activity regimes were characterized by distinct information coding properties in the model. These results provide a theoretical framework for explaining the origin and the information coding properties of the diverse non-rhythmic states of wakefulness.

## Results

### Recurrent Networks Exhibit a Spectrum of Asynchronous Regimes upon Modulation of Afferent Excitation

**Figure 1:**
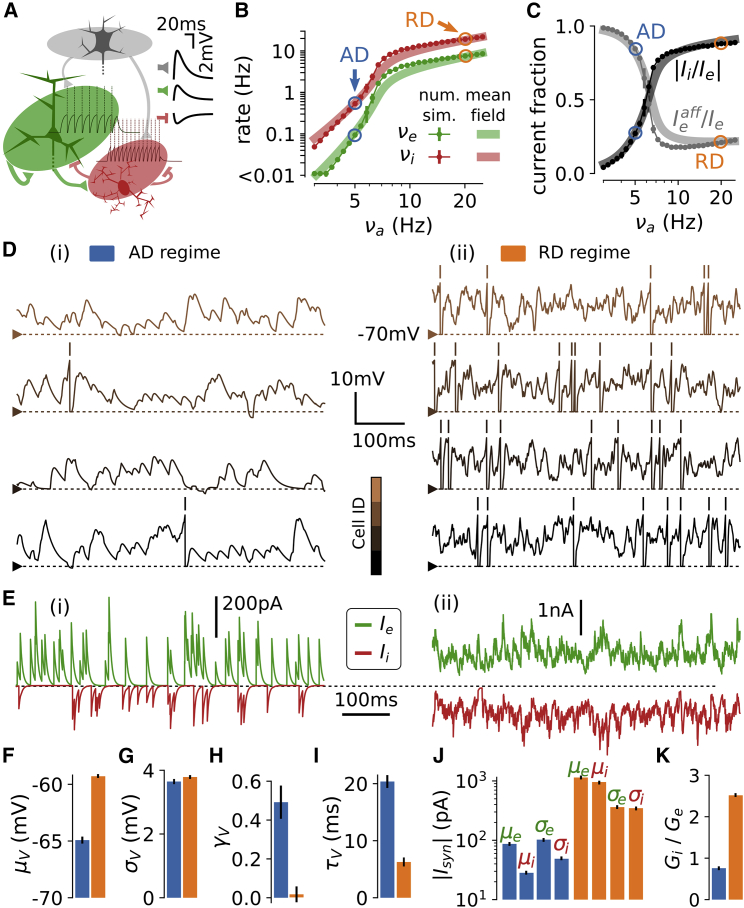
A Spectrum of Asynchronous Regimes in a Recurrent Spiking Network upon Variation of Afferent Excitation (A) Schematic of the model. An afferent excitatory input targets the recurrently connected excitatory (green) and inhibitory (red) populations. In the inset, post-synaptic deflections at $V_{m}$ = −70 mV associated with each type of synaptic connection (gray, afferent population). The spiking response of single neurons to a current pulse of 120 pA is shown in the background. (B) Stationary firing rates of the excitatory (green) and inhibitory (red) populations as a function of $\nu_{a}$ (dots with error bars represent mean ± SEM over n = 10 simulations). The AD (blue circle) and the RD (orange circle) levels are indicated. The mean-field predictions (thick transparent lines) are also shown. (C) Fraction of afferent currents within the sum of recurrent and afferent excitatory currents (${\text{I}}_{\text{e}}^{\text{aff}}$/${\text{I}}_{\text{e}}$, gray) and absolute ratio between inhibitory and excitatory currents ($\left|{\text{I}}_{\text{i}}/{\text{I}}_{\text{e}}\right|$, black) as a function of $\nu_{a}$ (n = 10 simulations; thick transparent lines: mean-field predictions). (D) Membrane potential traces for four neurons in the AD (i) and in the RD (ii) regimes. (E) Excitatory (green) and inhibitory (red) synaptic currents targeting a single neuron in the AD (i, left) and RD (ii, right) regimes. (F–K) Mean depolarization $\mu_{\text{v}}$ (F), standard deviation σ_V_, (G), skewness of the $V_{m}$ distribution $\gamma_{\text{v}}$ (H), autocorrelation time ${\text{τ}}_{\text{V}}$ (I), mean and SD (μ and σ, respectively) of the excitatory (indexed by e) and inhibitory currents (indexed by i) over time (J), and ratio of inhibitory to excitatory synaptic conductances ${\text{G}}_{\text{i}}$/${\text{G}}_{\text{e}}$ (K). Values are evaluated on excitatory cells on a single simulation, error bars represent variability (SD) over n = 10 cells. See also Figures S1 and S2 and Table S1.

We hypothesized that the non-rhythmic regimes of wakefulness could be described by a set of emergent solutions of recurrent activity in excitatory and inhibitory spiking networks. Specifically, we reasoned that regimes of intense synaptic activity (Brunel, 2000, Kumar et al., 2008, Renart et al., 2010, van Vreeswijk and Sompolinsky, 1996) should be complemented with regimes of low spiking, where single-neuron dynamics is driven by a few synaptic events, to describe the lower depolarization that characterizes asynchronous regimes associated with moderate arousal (McGinley et al., 2015a). Consequently, we explored the dynamics of spiking networks in a wide range of recurrent activity, down to recurrent activity lower than 0.1 Hz. We implemented a randomly connected recurrent network of leaky integrate-and-fire excitatory and inhibitory neurons with conductance-based synapses (Kumar et al., 2008). The network had the following experimentally driven features: recurrent synaptic weights leading to post-synaptic deflections below 2 mV at rest (Jiang et al., 2015, Lefort et al., 2009, Markram et al., 2015), probabilities of connections among neurons matching the relatively sparse ones observed in the adult mouse sensory cortex (Jiang et al., 2015), an afferent input describing the synchronized excitatory thalamic drives onto sensory cortices (Bruno and Sakmann, 2006), and a higher excitability of inhibitory cells to model the high firing of the fast-spiking non-adapting interneurons (Markram et al., 2004). The network model is schematized in Figure 1A. All parameters are listed in Table S1.

We analyzed the emergent network dynamics as a function of the stationary level of afferent excitation. We found stable asynchronous dynamics over a wide range of excitatory and inhibitory activity. The stationary spiking of the network spanned four orders of magnitude (Figure 1B), while pairwise synchrony remained one order of magnitude below classical synchronous regimes (SI < 5e−3; see Figure S1 for a detailed analysis of the network’s residual synchrony). Varying the model’s afferent activity, $\nu_{a}$, from $\nu_{a}$ = 3 Hz to $\nu_{a}$ = 25 Hz resulted in a logarithmically graded increase of excitatory firing rates, $\nu_{e}$, from $\nu_{e}$ = 0.004 Hz to $\nu_{e}$ = 8.5 Hz and inhibitory firing rates, $\nu_{i}$, from $\nu_{i}$ = 0.07 Hz to $\nu_{i}$ = 21.8 Hz (Figure 1B). Thus, recurrent dynamics exponentiated the level of afferent input. Importantly, the relative contributions of the afferent and recurrent excitation in shaping the single-neuron dynamics varied over the different levels of activity (gray curve in Figure 1C). It varied from regimes dominated by the afferent excitation ($I_{e}^{aff}/I_{e}$ > 0.75 for $\nu_{a}\leq \;$6 Hz, where $I_{e}$ is the sum of the afferent $I_{e}^{aff}$ and recurrent $I_{e}^{rec}$ excitatory currents) to a recurrent connectivity-dominated regime ($I_{e}^{rec}/I_{e}$ > 0.73 for $\nu_{a}$ ≥ 12 Hz). The ratio between mean inhibitory and excitatory synaptic currents ($I_{i}$/$I_{e}$, where $I_{i}$ is the recurrent inhibitory current) varied over those different activity levels (black curve in Figure 1C). It gradually varied from excitatory-dominated regimes where $I_{i}$/$I_{e}\ll \;$1 ($I_{i}$/$I_{e}$ < 0.50 below for $\nu_{a}$ = 6 Hz) to balanced activity where $I_{i}$/$I_{e}∼1$ ($I_{i}$/$I_{e}$ > 0.85 for $\nu_{a}$ ≥ 12 Hz). We refer to this continuum of diverse emergent solutions of recurrent activity as a “spectrum” of asynchronous regimes.

We selected two levels of afferent drive leading to two relative extreme states along this spectrum (Figure 1B). The first example, termed the afferent input-dominated state (AD), was a state found at low afferent excitation that was characterized by temporally sparse spiking activity and was dominated by its afferent excitation (see below). The second example state, termed the recurrent input-dominated state (RD), was found at high afferent excitation that was characterized by temporally dense spiking activity and was dominated by its synaptically balanced recurrent activity (see below). We show samples of membrane potential traces (Figure 1D) and synaptic currents (Figure 1E) for the two selected regimes.

**Figure 2:**
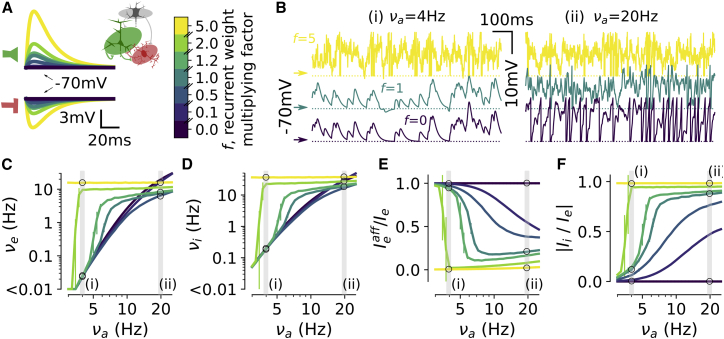
The Spectrum Is Conditioned to Moderate Strength of Recurrent Interactions (A) Post-synaptic deflections following an excitatory (top) and inhibitory (bottom) event as a function of the modulating factor for synaptic weights (color-coded scale on the right). (B) Sample traces of activity at low (left, i, ${\text{ν}}_{\text{a}}$ = 4 Hz) and high (right, ii, ${\text{ν}}_{\text{a}}$ = 20 Hz) levels of afferent activity for different strength of synaptic weights (same color code as in A). (C–F) Excitatory stationary firing rates (${\text{ν}}_{\text{e}}$ in C), inhibitory stationary firing rates (${\text{ν}}_{\text{i}}$ in D), fraction of afferent excitatory current (${\text{I}}_{\text{e}}^{\text{aff}}$/${\text{I}}_{\text{e}}$ in E), and inhibitory to excitatory current ratio ($\left|{\text{I}}_{\text{i}}/{\text{I}}_{\text{e}}\right|$ in F) as a function of ${\text{ν}}_{\text{a}}$ for different factors of recurrent synaptic weights. Data are presented as mean ± SEM over n = 10 simulations. Non-visible error bars correspond to variabilities smaller than the marker size. See also Figure S3.

For high afferent excitation ($\nu_{a}$ = 20 Hz, RD), the recurrent activity was dense (> 1 Hz, here $\nu_{e}$ = 7.6±0.1 Hz and $\nu_{i}$ = 19.2±0.2Hz), and the network displayed balanced asynchronous dynamics characterized by (1) mean depolarized $V_{m}$ ($\mu_{V}\;$ = −59.3 + 0.1 mV; Figure 1F) with standard deviation $\sigma_{V}\;$ = 3.7±0.1 mV (Figure 1G), which implied $V_{m}$ fluctuations being closer to the spiking threshold (see $V_{m}$ traces in Figure 1D); (2) symmetric $V_{m}$ distribution (Figure 1H; skewness $\gamma_{V\;}$ = 0.02±0.04), a signature of Gaussian fluctuations (coefficient of determination of a Gaussian fitting after blanking spikes: $R^{2}\;$ = 0.994±0.002); (3) fast membrane potential fluctuations (autocorrelation time $\tau_{V}$ = 6.2±0.8 ms, much lower than the membrane time constant at rest $\tau_{\;m}^{0}\;$ = 20 ms; Figure 1I); (4) high conductance state (synaptic conductances sum up to more than four times the leak conductance [Destexhe et al., 2003], conductance ratio was 6.6±0.1); (5) balanced excitatory and inhibitory currents ($\left|I_{i}/I_{e}\right|$ = 0.881±0.003; Figure 1C) with large means compared to their temporal fluctuations ($\mu_{e}$/$\sigma_{e}\;$ = 3.2±0.1 and $\mu_{i}$/$\sigma_{i}\;$ = 2.8±0.1; Figure 1J); and (6) the predominance of the recurrent activity in shaping single-neuron dynamics (recurrently mediated synaptic currents were 84.6% ± 0.1% of the membrane currents, afferent excitatory currents were 10.8%± 0.1% and leak currents 4.6% ± 0.1%).

**Figure 3:**
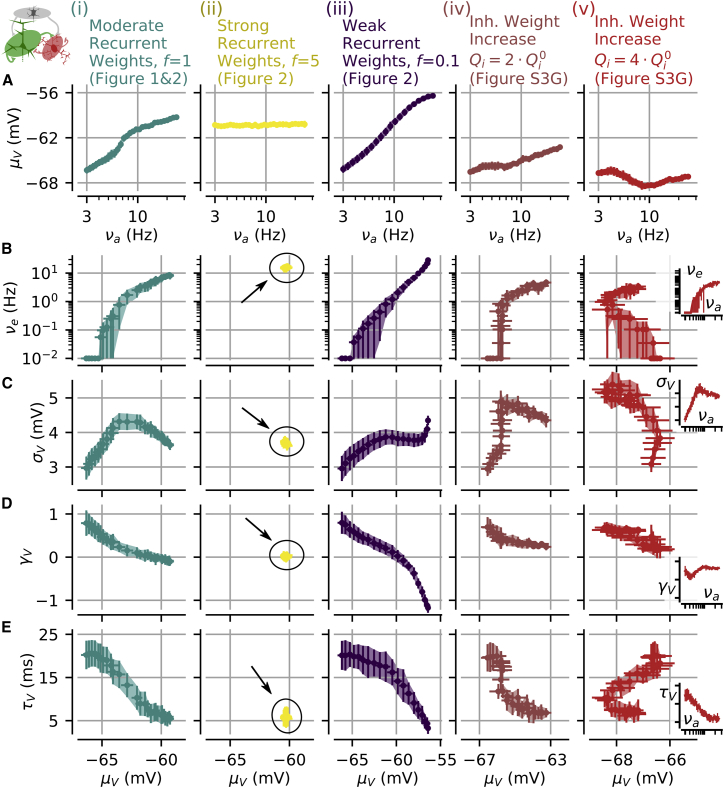
Relationship between Membrane Potential Features and Firing Rates for Different Parameter Settings of the Recurrent Network (A) Relationship between $\nu_{a}$ and $\mu_{V}\;$ for a model network with different parameter settings. Specifically, (i) moderate and balanced recurrent interactions (the architecture shown in Figures 1 and 2), (ii) strong and balanced recurrent interactions for f = 5 in Figure 2, (iii) weak and balanced recurrent interactions found for f = 0.1 in Figure 2, (iv) a moderately inhibitory-augmented case with $Q_{i}$ = 2$\cdot Q_{i0}$, and (v) a strongly inhibitory-augmented case with $Q_{i}$ = 4$\cdot Q_{i0}$. (B) Relationship between $\mu_{V}\;$ and $\nu_{e}$ in the different cases shown in (A). (C) Relationship between $\mu_{V}\;$ and $\sigma_{V}\;$ under the different scenarios shown in (A). (D) Relationship between $\mu_{V}\;$ and ${\text{γ}}_{\text{V}}$. (E) Relationship between $\mu_{V}\;$ and $\tau_{V}$. The mean ± SEM over 10 excitatory cells averaged over 10 network simulations lasting 1 s each is presented. The insets in (B)–(E) (v) show the dependency of ${\text{ν}}_{\text{e}}$, ${\text{σ}}_{\text{V}}$, ${\text{γ}}_{\text{V}}$, and ${\text{τ}}_{\text{V}}$on ${\text{ν}}_{\text{a}}$. See also Figure S4.

**Figure 4:**
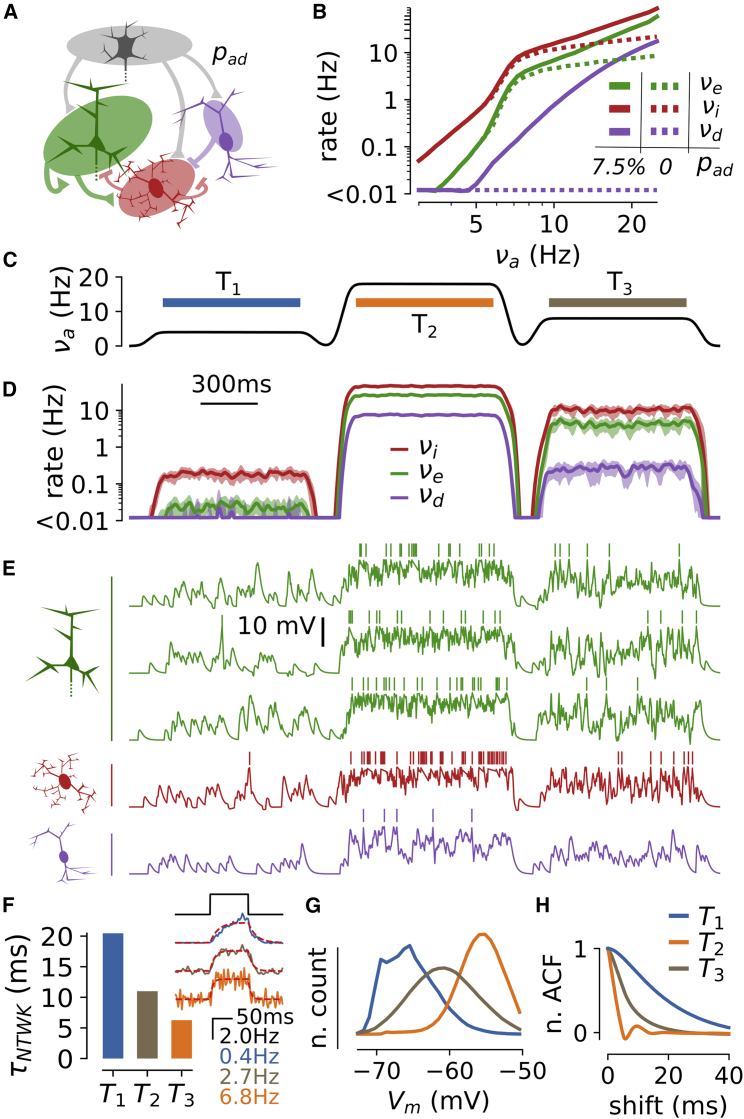
Modulation of Network Activity upon a Time-Varying Afferent Excitation (A) Schematic of the network model including the disinhibitory circuit. The parameter ${\text{p}}_{\text{ad}}$ corresponds to the connection probability between the afferent and disinhibitory populations. (B) Stationary co-modulations of the excitatory (${\text{ν}}_{\text{e}}$, green), inhibitory (${\text{ν}}_{\text{i}}$, red), and disinhibitory (${\text{ν}}_{\text{d}}$, purple) rates in absence (${\text{p}}_{\text{ad}}=0$, dashed line; reproduced from Figure 1B) and in the presence of a disinhibitory circuit (solid line, ${\text{p}}_{\text{ad}}=7.5\text{\%}$). (C–E) Network dynamics in response to a time-varying input. (C) Waveform for the afferent excitation. (D) Temporal evolution of the instantaneous firing rates (binned in 2-ms windows and smoothed with a 10-ms-wide Gaussian filter) of the excitatory (${\text{ν}}_{\text{e}}$, green), inhibitory (${\text{ν}}_{\text{i}}$, red), and disinhibitory (${\text{ν}}_{\text{d}}$, purple) populations. Mean ± SEM over n = 10 trials. Time axis as in (C). (E) Membrane potential traces in a trial (green, excitatory cells; red, inhibitory cell; purple, disinhibitory). To highlight mean depolarization levels, the artificial reset and refractory mechanism has been hidden by blanking the 10 ms following each spike emission (see also Figure S6). Time axis as in (C). (F) Network time constants ${\text{τ}}_{\text{NTWK}}$ for the three different levels of afferent activity considered in (C) (blue, ${\text{ν}}_{\text{a}}$ = 4 Hz; orange, ${\text{ν}}_{\text{a}}$ = 18 Hz; brown, ${\text{ν}}_{\text{a}}$ = 8 Hz). The time constant was determined by stimulating the network with a 100-ms-long step input of afferent activity of 2 Hz (black curve in the inset) and fitting the trial-average responses with an exponential rise-and-decay function (red dashed curves, see STAR Methods). We show the average over 100 stimulus repetitions of the network responses in the inset. (G and H) Pooled membrane potential histograms for the three different stimulation periods (G) and pooled normalized autocorrelation functions (H). Data were obtained by pooling together the $V_{m}$ after blanking spikes over 100 excitatory neurons in each interval for a single network simulation. See also Figure S5 and Table S2.

For low afferent activity ($\nu_{a}$ = 5 Hz, AD), asynchronous dynamics exhibited a qualitatively different set of electrophysiological features. Spiking activity was sparse ($\nu_{e}$ = 0.09±0.01 Hz and $\nu_{i}$ = 0.54±0.0143 Hz; Figure 1B), which, at the single-neuron level, was associated with (1) a longer distance between the mean *V*_*m*_ and the spiking threshold ($\mu_{V}\;$ = 64.1±0.3 mV; Figure 1F); (2) a strongly skewed *V*_*m*_ distribution ($\gamma_{V\;}$ = 0.49±0.09; Figure 1H); (3) slower $V_{m}$ fluctuations ($\tau_{V}$ = 20.4±1.1 ms; Figure 1I); (4) a lower conductance state preserving the efficacy of synaptically evoked depolarizations (synaptic conductances increased the input conductance by only 18.2%±1.0%); (5) excitatory-dominated synaptic currents, where the mean of the excitatory currents largely exceeded those of inhibitory currents ($\left|I_{i}/I_{e}\right|$ = 0.28±0.02, Figure 1C), leading to a nearly unitary ratio of conductances ($G_{i}$/$G_{e}\;$ = 0.8±0.2, instead of $G_{i}$/$G_{e}=\;$2.5±0.1 for the balanced currents of RD; Figure 1K); and (6) the predominance of the non-recurrent components in shaping single-neuron dynamics (recurrently mediated synaptic currents were 14.6% ± 0.2% of the membrane currents, afferent excitatory current were 44.9%± 1.3%, and leak current contributions were 40.4% ± 1.5%). In contrast to the RD state, the stability of the AD regime did not rely on the balance between excitatory and inhibitory synaptic currents (see Figure 1E): the low amount of recurrent inhibitory currents did not cancel the afferent-dominated excitatory currents (see Figures 1C, 1E, and 1J). Rather, leak currents ensured stability by significantly contributing to single-neuron integration: the temporal dynamics of the membrane potential was dominated by leak-mediated repolarization following sparse synaptic events (see Figure 1D) and, accordingly, $\tau_{V}$ = 20.4±1.1 ms was close to the membrane time constant at rest $\tau_{m}^{0}$ = 20 ms.

### A Mean-Field Description Predicts the Emergence of the Spectrum

To understand whether the variations of the firing rates ($\nu_{e}$,$\;\nu_{i}$) and the $V_{m}$ fluctuations properties $\left(\mu_{V},\;\sigma_{V},\tau_{V},\;\gamma_{V}\right)$ are sufficient for the emergence of the spectrum, we developed and analyzed a “mean field” description of network activity including these quantities (see STAR Methods). The mean-field description of network activity reduces the firing rate dynamics of each population into the dynamics of a prototypical neuron whose behavior is captured by a rate-based input-output function (Renart et al., 2004). In the mean-field approach, the neuronal input-output function is determined by converting the input firing rates into Gaussian fluctuations of synaptic currents, which are in turn translated into an output firing rate using estimates from stochastic calculus (Tuckwell, 2005). Building on previous work (Zerlaut et al., 2016), we extended this formalism so that the input firing rates are converted into $V_{m}$ fluctuations properties that also include higher-order non-Gaussian properties (such as γ_V_ and the tail integral of the distribution) and that are converted into an output firing rate with a semi-analytical approach (see STAR Methods). We found that the spectrum of dynamics found in the numerical simulations was also present in such a mean-field description (Figures 1B, 1C, and S2). Because the mean-field description only considered $\nu_{e}$,$\;\nu_{i}$, $\mu_{V},\;\sigma_{V},\tau_{V},\;\gamma_{V}$, this analysis further demonstrates that changes in those parameters are sufficient to generate changes in the spectrum. This confirms that the spectrum can be generated also without relying on specific details of numerical networks (such as a degree of clustering within the drawn connectivity) or more complex dynamical features (such as pairwise synchrony, or deviations from the Poisson spiking statistics), which were not included in the mean-field approach.

### Moderate Strength of Recurrent Interactions Is Necessary for the Emergence of the Spectrum

What are the crucial parameters that lead to the emergence of the spectrum of activity states? We addressed this question through parameter variations in the numerical model.

We first considered what happened when increasing, with respect to the reference network configuration considered above, the value of the recurrent synaptic weights (Figure 2). We modulated both the excitatory and inhibitory synaptic weights using a common factor *f* (see Figure 2A) to keep a balanced setting between excitation and inhibition across the considered levels of recurrent interactions. This prevented the emergence of highly synchronized regimes (Brunel, 2000); see Figure S3. When multiplying recurrent weights by a moderate f value with f in the range between 0.1 and 2 (see f = 0.5 and f = 1.2 in Figure 2A), the network was still able to create states of very low (respectively, very high) activity at lower (respectively, higher) afferent activity. Under these conditions, the network exponentiated the level of afferent input to generate recurrent activity spanning several orders of magnitude (from 0.004 to 15 Hz; Figures 2C and 2D) with transitions from regimes dominated by afferent inputs ($I_{e}^{aff}$/$I_{e}$ > 0.75 for $\nu_{a}$ < 5 Hz; Figure 2E) and excitatory currents ($\left|I_{i}/I_{e}\right|$ < 0.25 for $\nu_{a}$ < 5 Hz; Figure 2F) to regimes dominated by recurrent activity ($I_{e}^{aff}$/$I_{e}$ < 0.5 for $\nu_{a}$ > 20 Hz; Figure 2E) and balanced synaptic currents ($\left|I_{i}/I_{e}\right|$ > 0.75 for $\nu_{a}$ > 20 Hz; Figure 2F). Thus, the network displayed the spectrum of activity regimes over the entire range for 0.1 < f < 2. However, when *f* > 2 (see example of f = 5 in Figure 2A), the network generated states of dense balanced activity throughout the entire range of afferent input rates (see yellow curves in Figures 2C–2F) and showed a small range of variations of recurrent firing rates (15–17 Hz; yellow curve in Figure 2C). For very low values of the f factor (see f = 0, dark purple curves in Figure 2), the population only displayed regimes dominated by afferent inputs ($I_{e}^{aff}$/$I_{e}$ = 1; Figure 2E) and excitatory currents ($\left|I_{i}/I_{e}\right|$ = 0; Figure 2F). Consistent with the need of moderate recurrent interactions, increasing recurrent connectivity with respect to the reference scenario by augmenting connection probability ($p_{conn}\;$ > 20%) restricted the occurrence of AD-type activity to lower and lower afferent activity levels (see Figure S3B).

Other experimentally driven constraints of network implementation were less critical for generating the spectrum. Varying synaptic weights of the afferent input in the [−50%, +50%] range shifted the onset of the activity increase (in terms of the $\nu_{a}$ level) but allowed for a set of asynchronous regimes across orders of magnitude (Figure S3C). Varying the inhibitory excitability by shifting the spiking threshold in the [−57, −52] mV range also did not affect the ability of the network to display the spectrum (Figure S3D). This was also the case when varying the network size (Figure S3E) and the excitatory and inhibitory synaptic weights independently in the [−50%, +50%] range (Figures S3F and S3G). However, more extreme variations (very low inhibitory excitabilities $V_{thre}^{inh}\geq$ −51 mV [Figure S3E], strong excitatory weights $Q_{e}\geq$ 4 nS [Figure S3F], and weak inhibitory weights $Q_{i}\leq$ 5 nS [Figure S3G]) led to a recurrent network with a very strong excitatory-to-excitatory loop and produced saturated ($\nu_{e}$ = $\nu_{i}$ = 200 Hz) and highly synchronized (SI > 0.9) activity because of weak inhibition unable to prevent an excitatory runaway (Brunel, 2000). Low afferent input weights $Q_{a}\leq$ 1 nS also prevented the appearance of the spectrum because only quiescent regimes ($\nu_{e}$ = $\nu_{i}$ = 0 Hz) could be observed in the $\nu_{a}$ < 25 Hz range of afferent inputs (Figure S3C).

### Relationships between Afferent Activity, $V_{m}$ Properties, and Firing Rate

We computed (Figure 3) the firing rate ${\text{ν}}_{\text{e}}$ and ${\text{μ}}_{\text{V}}$, ${\text{σ}}_{\text{V}}$, ${\text{γ}}_{\text{V}}$, and ${\text{τ}}_{\text{V}}$ in excitatory cells under the following five different conditions: (1) balanced and moderate recurrent interactions (f *= 1*) corresponding to a set of parameters that generates the spectrum of asynchronous states, (2) balanced and strong recurrent interactions (f = 5); (3) balanced and weak recurrent interactions (f = 0.1), (4) weakly inhibitory-augmented recurrent interactions (inhibitory synaptic weights increased by a factor of 2 with respect to the values of case 1 and Table S1), and (5) strongly inhibitory-augmented recurrent interactions (inhibitory synaptic weights increased by a factor of 4 with respect to the values of case 1 and Table S1). We found that the first four cases showed a monotonic relationship between ${\text{ν}}_{\text{a}}$ and ${\text{μ}}_{\text{V}}$ (Figure 3A). This observation allowed us to invert the ${\text{ν}}_{\text{a}}$ versus ${\text{μ}}_{\text{V}}$ relationship. Therefore, ${\text{μ}}_{\text{V}}$ can be used as a proxy of ${\text{ν}}_{\text{a}}$ and this allowed us to study the dependence of ${\text{ν}}_{\text{e}}$ (Figure 3B) and other membrane potential properties (Figures 3C and 3E) on ${\text{μ}}_{\text{V}}$.

We then considered the relationship between ${\text{μ}}_{\text{V}}$ and ${\text{ν}}_{\text{e}}$ and the relationships between ${\text{μ}}_{\text{V}}$ and ${\text{σ}}_{\text{V}}$, ${\text{γ}}_{\text{V}}$, and ${\text{τ}}_{\text{V}}$. We first studied the case of moderate (*f = 1*; column i in Figure 3B) strength of the recurrent connectivity. We found that the relationship between ${\text{μ}}_{\text{V}}$ and ${\text{ν}}_{\text{e}}$ was monotonic, with ${\text{μ}}_{\text{V}}$ varying over a range of several millivolts and ${\text{ν}}_{\text{e}}$ spanning more than three orders of magnitude. For the relationship between ${\text{σ}}_{\text{V}}$ and ${\text{μ}}_{\text{V}}$, we found a non-monotonic, inverted-U-shaped, relationship (Figure 3Ci) compatible with the following scenario (see Kuhn et al., 2004). At hyperpolarized levels, $\sigma_{V}\;$ increased with $\mu_{V}\;$ because an increase in $\mu_{V}\;$ is associated with an increase in the afferent and recurrent frequencies (Figures 3A and 3B) and, as a general phenomenon, an increase in synaptic events per unit time (here, $\nu_{a}$, $\nu_{e}$, and $\nu_{i}$) results in an increase in the amplitude of the $V_{m}$ fluctuations (here, $\sigma_{V}\;$) at a fixed size of synaptic events (Daley and Vere-Jones, 2003). However, this trend competed with the shunting effect associated with increasing synaptic activities (Chance et al., 2002, Destexhe et al., 2003). At depolarized levels, the high levels of synaptic activity (Figures 3A and 3B) led to high membrane conductance and caused a strong shunting that dampened the size of post-synaptic events, thus decreasing the fluctuations amplitude $\sigma_{V}\;$ despite the increase in event frequencies (Kuhn et al., 2004). The constraints to $V_{m}$ fluctuations due to the spiking threshold and reversal potentials made a weak contribution to the observed $\mu_{V}\;$-$\sigma_{V}\;$ relationship (Kuhn et al., 2004); see Figure S5. The ${\text{γ}}_{\text{V}}$-${\text{μ}}_{\text{V}}$ relationship showed a gradual decay with ${\text{μ}}_{\text{V}}$ (Figure 3Di), starting from positive skewness $\left({\text{γ}}_{\text{V}}∼\;1\right)$ for low ${\text{μ}}_{\text{V}}$ values toward ${\text{γ}}_{\text{V}}$ ∼0 (corresponding to a symmetric distribution) for higher ${\text{μ}}_{\text{V}}$ values most likely because, at high levels of synaptic events, the statistical moments beyond second order vanish (Daley and Vere-Jones, 2003). Finally, we observed a monotonically decreasing relationship between ${\text{τ}}_{\text{V}}$ and ${\text{μ}}_{\text{V}}$ (Figure 3Ei), in agreement with the observation that single-neuron integration is faster because of an increase of synaptically mediated membrane conductance (Destexhe and Paré, 1999).

The relationships between ${\text{μ}}_{\text{V}}$, ${\text{ν}}_{\text{e}}$, ${\text{σ}}_{\text{V}}$, ${\text{γ}}_{\text{V}}$, and ${\text{τ}}_{\text{V}}$ observed for balanced and moderate recurrent connectivity across regimes were not found to be all conserved in conditions in which the single-neuron parameters were the same but the network parameter settings varied. In the case of strong synaptic weights (column ii in Figure 3 and f = 5 in Figure 2), the set of emergent solutions was confined in a narrow region of network activity resulting in ${\text{ν}}_{\text{e}}$ spanning less than an order of magnitude (Figure 3B) and approximately constant $V_{m}$ fluctuations properties across regimes (Figures 3C–3E; ${\text{μ}}_{\text{V}}∼$−60 mV, ${\text{σ}}_{\text{V}}∼$3.7 mV, ${\text{γ}}_{\text{V}}∼$0, and ${\text{τ}}_{\text{V}}∼$5 ms). For weak recurrent interactions (column iii in Figure 3 and f = 0.1 in Figure 2), the set of regimes contrasted with the moderate interaction case because of the increasing relationship between $\mu_{V}\;$ and $\sigma_{V}\;$ (Figure 3C) and a range of ${\text{γ}}_{\text{V}}$ characterized by large variations down to very negative values (Figure 3D). On the other hand, the $\mu_{V}\;$-$\nu_{e}$ and $\mu_{V}\;$-$\tau_{V}$ relationships were similar between the weak (f = 0.1) and moderate (f = 1) recurrence cases (Figures 3B and 3E). In the case of strongly inhibitory-augmented architecture (column v and red curves in Figure 3), the monotonic increase between ${\text{ν}}_{\text{a}}$ and ${\text{μ}}_{\text{V}}$ was not observed and thus ${\text{μ}}_{\text{V}}$ could not be used as a proxy for ${\text{ν}}_{\text{a}}$. As a consequence, even if this latter case showed some features of the spectrum of asynchronous states as a function of ${\text{ν}}_{\text{a}}$ (Figure 3v; log-distributed ${\text{ν}}_{\text{e}}$, inverted-U shape for ${\text{σ}}_{\text{V}}$, and decreasing ${\text{τ}}_{\text{V}}$), the relationship between ${\text{ν}}_{\text{e}}$, membrane potential features and ${\text{μ}}_{\text{V}}$ displayed very different behavior compared to that of the balanced network with moderate connectivity strength (Figure 3i). In case v, the ${\text{μ}}_{\text{V}}$-${\text{ν}}_{\text{e}}$ and ${\text{μ}}_{\text{V}}$-${\text{τ}}_{\text{V}}$ relationship displayed C-shape curves (red curves in Figures 3B and 3E) rather than the monotonic relationships observed in case i (blue curves in Figures 3B and 3E) and the ${\text{μ}}_{\text{V}}$-${\text{σ}}_{\text{V}}$ relationship corresponded to a steeply decreasing relationship. The case of moderately inhibitory-augmented recurrent architecture (column iv in Figure 3) provided an intermediate case between case v and case i.

These results demonstrate that the relationships described above are not only determined by single cell properties (which were kept constant across comparisons in Figure 3), but they are strongly shaped by how network properties constrain the emergent activity regimes and their associated inputs to a neuron.

### Disinhibition Broadens the Spectrum

To test the generality of the findings, we considered a more complex network including a disinhibitory circuit (see Figure 4A and Table S2). The disinhibitory cells formed inhibitory synapses on inhibitory neurons. Because experimental evidence suggests weak inputs into disinhibitory cells from the local network (Jiang et al., 2015, Pfeffer et al., 2013), we assumed in the model that the disinhibitory cells received only excitatory afferent inputs. By lowering the excitability of the inhibitory population as a function of ${\text{ν}}_{\text{a}}$, the disinhibitory activity allowed excitatory-dominated states to span higher ranges of firing rate values (up to ${\text{ν}}_{\text{e}}$ = 58.3 ± 3.9 Hz for ${\text{ν}}_{\text{a}}$ = 25 Hz; see Figure 4B) while remaining largely asynchronous (SI < 0.12; Figure S3I). As the model configuration inclusive of the disinhibitory circuit presumably provided a more realistic setting, we hereafter continued our analysis using the three-population model of Figure 4A.

### Modulation of the Network State upon Time-Varying Afferent Excitation

We studied whether the model could generate the spectrum of states with time-varying inputs. Given that in awake cortical data different states can persist for time scales of < 1 s (McGinley et al., 2015a), we focused on studying network dynamics when inputs were stationary for hundreds of milliseconds. We stimulated the three-population model (Figure 4C) with a time-varying waveform made of three 900-ms-long plateaus of presynaptic activity at low ($\nu_{a}$ = 4 Hz, $T_{1}$ period, blue interval), high ($\nu_{a}$ = 18 Hz, $T_{2}$, orange), and intermediate ($\nu_{a}$ = 8 Hz, $T_{3}$, gray) levels. Figure 4D shows the temporal evolution of the firing rates (averaged over n = 10 trials) and Figure 4E shows the $V_{m}$ dynamics in the three cellular populations included in the model in a single trial. We observed dynamic modulations of the firing rate with time scales to reach stationary behavior that were similarly fast across the different cell types (red, green, and purple in Figure 4D). We found the relaxation time of the network ($\tau_{NTWK}$, estimated by fitting the response to a short step of afferent activity; see Figure 4F) to be between 4 and 20 ms (with a monotonic dependence on the level of ongoing activity, as predicted theoretically [Destexhe et al., 2003, van Vreeswijk and Sompolinsky, 1996]). For time scales longer than few hundred milliseconds, the network dynamics can thus be considered as stationary. Consequently, the characterization described above for stationary states (Figures 1 and 4B) should also hold when the analysis was restricted to the three separate windows $T_{1}$, $T_{2}$, and $T_{3}$ (see Figures 4C–4E). Indeed, the first period ($T_{1}$; blue in Figures 4C and 4F–4H) displayed the properties of the AD regime with its low firing rate ($\nu_{e}$ = 0.02 ± 0.01 Hz) and slow ($\tau_{V}$ = 17.8 ± 0.3 ms), skewed ($\gamma_{V\;}$ = 0.55 ± 0.01), and hyperpolarized ($\mu_{V}\;$ = −65.3 ± 0.1 mV) $V_{m}$ fluctuations. Similarly, the second period ($T_{2}$; orange in Figure 4) displayed the properties of the AD regime with its high rate ($\nu_{e}$ = 25.9 ± 0.6 Hz) and its fast ($\tau_{V}$ = 2.3 ± 0.1 ms), depolarized ($\mu_{V}\;$ = −55.9 ± 0.1 mV), and Gaussian ($R^{2}\;$ = 0.99 ± 0.01, Gaussian fitting after blanking spikes) $V_{m}$ fluctuations. The third period ($T_{3}$; gray in Figure 4) displayed the properties of an intermediate regime (see Figure 3) with $\nu_{e}$ = 4.2 ± 0.3 Hz, $\mu_{V}\;$ = −60.8 ± 0.2 mV, $\sigma_{V}\;$ = 4.3 ± 0.1 mV, and $\tau_{V}$ = 6.8 ± 0.3 ms.

### Recordings in the S1 Cortex of Awake Mice Confirm Model Predictions

**Figure 5:**
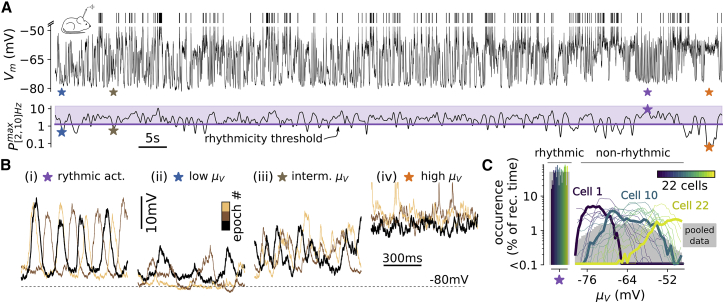
In the S1 Cortex of Awake Mice, Non-rhythmic Activity Is Associated with Various Membrane Depolarization Levels (A) Intracellular recordings of $V_{m}$ fluctuations (top) during spontaneous activity and maximum power of $V_{m}$ in the [2, 10]-Hz band (bottom). Three periods classified as non-rhythmic epochs (blue, brown, and orange stars) and one rhythmic epoch (purple star) are highlighted. Note the $Pow_{\left[2,10\right]\text{Hz}}^{\text{max}}$ index being below (for the three non-rhythmic events) and above (for the rhythmic event) the rhythmicity threshold. (B) $V_{m}$ sample epoch classified as (i) rhythmic, (ii) low ${\text{μ}}_{\text{V}}$ (${\text{μ}}_{\text{V}}$ < −70mV), (iii) intermediate ${\text{μ}}_{\text{V}}$(${\text{μ}}_{\text{V}}\in \;$[−70, −60] mV), and (iv) high ${\text{μ}}_{\text{V}}$ (${\text{μ}}_{\text{V}}$ > −60 mV). The black traces correspond to the prolonged epoch shown in (A) and the two other samples (copper colors) were extracted from the same intracellular recording. (C) Fraction of occurrence of the rhythmic and non-rhythmic epochs at their respective levels of mean depolarization, ${\text{μ}}_{\text{V}}$. Single-cell recordings have been sorted with respect to their average level of non-rhythmic activity ${\text{μ}}_{\text{V}}$ and color coded accordingly. Three cells—1, 22, and 10 (shown in A and B)—are highlighted. The plain gray area represents the dataset after pooling together all $V_{m}$ recordings (n = 22 cells). Note that the fraction of occurrence of rhythmic activity in the pooled data corresponds to 50% as a consequence of the definition of the rhythmicity threshold (see STAR Methods).

We performed intracellular patch-clamp recordings from layer 2/3 neurons of the S1 cortex of awake mice (n = 22 cells in n = 8 animals) during spontaneous activities (Figure 5). These recordings (Figure 5A) showed fluctuations in the membrane potential of the recorded cell between rhythmic and asynchronous dynamics as described in previous reports (Crochet and Petersen, 2006, Poulet and Petersen, 2008). Because our focus was on asynchronous cortical dynamics, we introduced a threshold in the low-frequency power of the $V_{m}$ recordings, called the rhythmicity threshold (see STAR Methods). We classified as rhythmic periods all the time stretches for which the $V_{m}$ power exceeded this threshold (Figure 5A) and we considered for further analyses only the epochs of network activity with $V_{m}$ power below this threshold (“non-rhythmic” stretches; see Figure 5A). Results were robust to variations of this threshold (Figure S6D).

We then divided the stretches of non-rhythmic activity into 500-ms-long epochs. Each epoch was considered a possible different state. We chose this epoch length as it offered a good compromise between the following constraints: (1) it was short enough to enable the identification of specific states of wakefulness (Figure S7) and (2) it was long enough to average synaptically driven $V_{m}$ dynamics and to analyze network activity beyond its own relaxation time constant (Reinhold et al., 2015). Similarly to previous findings in the auditory (McGinley et al., 2015b) and visual (Reimer et al., 2014) cortices of awake-behaving mice, we found non-rhythmic epochs of network activity in the S1 cortex that showed various levels of $\mu_{V}\;$. Figure 5B shows representative membrane potential epochs and their fraction of occurrence at the various $\mu_{V}\;$ levels over single cells (color coded in Figure 5C) and over the ensemble data (gray area in Figure 5C). Few cells (n = 3 out of 22, for example “cell 10” shown in Figures 5A and 5B) displayed non-rhythmic activity over a wide range of $\mu_{V}\;$ (> 20 mV). The majority of cells displayed non-rhythmic activity over a narrower range of $\mu_{V}\;$ (for the remaining n = 19 out of 22 the extent of $\mu_{V}\;$ was 10.8±4.1 mV; e.g., “cell 1” showed only hyperpolarized non-rhythmic activity and “cell 22” exhibited mostly depolarized non-rhythmic activity; see Figure 5C).

**Figure 6:**
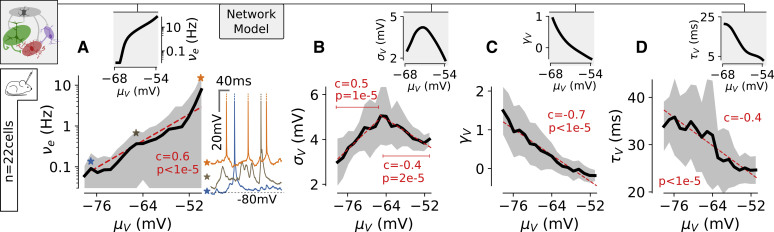
The Model Predicts the Electrophysiological Features Characterizing the Different Non-rhythmic Epochs of Wakefulness in the S1 Cortex (A) Spiking probability (${\text{ν}}_{\text{e}}$, in Hz) of intracellularly recorded layer 2/3 pyramidal cells within ${\text{μ}}_{\text{V}}$-classified epochs. The red dashed line is a linear regression between ${\text{μ}}_{\text{V}}$ and $\log_{10}\left({\text{ν}}_{\text{e}}\right)$ (see STAR Methods). The correlation coefficients and the p value of a one-tailed permutation test (see STAR Methods) are reported. In the right inset, we show 300-ms-long epochs displaying spikes for three levels of ${\text{μ}}_{\text{V}}$ (blue, brown, and orange stars in main plot). In the top inset, we show the predictions of the network model. (B) Co-modulation between ${\text{μ}}_{\text{V}}$ and ${\text{σ}}_{\text{V}}$. Note that the linear regression has been split into two segments (depicted in red) to test the significance of the non-monotonic relationship. (C) Co-modulation between ${\text{μ}}_{\text{V}}$ and ${\text{γ}}_{\text{V}}$. (D) Co-modulation between ${\text{μ}}_{\text{V}}$ and ${\text{τ}}_{\text{V}}$. See also Figures S6, S7, and S8.

One central prediction of the model was the occurrence of a range of different states at various $\mu_{V}\;$ values with ${\text{ν}}_{\text{e}}$ spanning over three to four orders of magnitude (inset in Figure 6A). This was confirmed in experimental data: hyperpolarized epochs displayed ${\text{ν}}_{\text{e}}<$ 0.1 Hz, while depolarized epochs exhibited ${\text{ν}}_{\text{e}}$ in the 10-Hz range (see Figure 6A). The wide range of ${\text{ν}}_{\text{e}}$ across the non-rhythmic states of wakefulness with different $\mu_{V}\;$ values was further confirmed by extracellular recordings (see Figure S8). We combined the previously described intracellular approach with extracellular recordings of the multiunit activity (MUA) in layer 2/3 (n = 4 mice, n = 14 cells; see STAR Methods). We found that the logarithm of the mean MUA within non-rhythmic epochs exhibited a robust linear correlation with $\mu_{V}\;$ (correlation coefficient c = 0.5; one-tailed permutation test: p < 1e−5; see Figure S8). This suggests that the wide range of rates predicted by the model was observed not only at the single-neuron level but also at the mass circuit activity level, as expected by the theoretical model.

Moreover, we measured in real data: (1) the standard deviation, $\sigma_{V}\;$; (2) the skewness of the $V_{m}$ distribution, $\gamma_{V\;}$; and (3) the speed of the $V_{m}$ fluctuations quantified by the autocorrelation time, $\tau_{V}$ (see STAR Methods). The network model predicted that (1) the $\sigma_{V}\;$-$\;\mu_{V}\;$ relationship should be non-monotonic with a peak in the intermediate $\mu_{V}\;$ range (inset of Figure 6B), (2) the $\gamma_{V\;}$-$\mu_{V}\;$ relationship should start from strongly positively skewed values $\left(\gamma_{V}∼\;1\right)$ and monotonically decrease with $\mu_{V}\;$ (inset of Figure 5 and Figure 6C), and (3) the $\tau_{V}$-$\mu_{V}\;$ relationship should be monotonically decreasing with a near-15-ms drop in $\tau_{V}$ (inset of Figure 6D). Remarkably, we found all those features in our experimental recordings (Figures 6B–6D). Moreover, those relationships were found to be highly significant (p < 5e−5 for all relationships; see Figures 6B–6D). The model prediction of a transition toward Gaussian fluctuations at high $\mu_{V}\;$ (Figure 4F) was also found to hold on real data: we fitted the pooled distributions with a Gaussian curve (see STAR Methods) and the coefficient of determination was $R^{2}\;$ = 0.99 ± 0.01 above $\mu_{V}\;$ = −60 mV compared to $R^{2}\;$ = 0.96 ±0.04 below $\mu_{V}\;$ = −60 mV (n = 55 $\mu_{V}\;$-defined distributions across 13 cells for $\mu_{V}\;$ > −60 mV and n = 129 $\mu_{V}\;$-defined distributions across the 22 cells for $\mu_{V}\;\leq \;$−60 mV; p = 3.2e−5, unpaired t test).

### Activity Levels along the Spectrum Have Different Computational Properties

**Figure 7:**
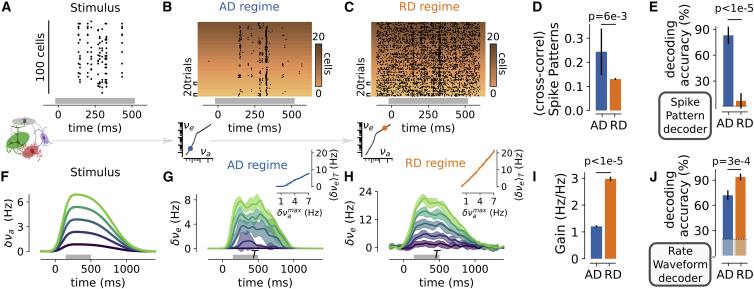
The AD Regime Enables the Precise Encoding of Complex Patterns of Presynaptic Activity, while the RD Regime Exhibits High Population Responsiveness to Afferent Inputs (A) Representative example of a presynaptic activity pattern that corresponds to 10 activations of different groups of 10 synchronously spiking units (randomly picked within the 100 cells of the presynaptic population) in a 500-ms window (see STAR Methods). (B) Spiking response of a sub-network of neurons (20 cells) across 20 trials in the AD regime. The y axis indexes both the neuron identity (color coded) and the trial number (vertical extent on a given color level). (C) Same as in (B) but for the RD regime. (D) Mean cross correlation of the output spiking patterns across realizations for a given input pattern (mean ± SEM over 10 input patterns; for each input pattern we computed the mean cross correlation across all pairs of observations of the 20 realizations; two-sided Student’s t test). (E) Performance in decoding the pattern identity from the sub-network spiking patterns with a nearest-neighbor classifier (see STAR Methods). The mean accuracy ± SEM over 10 patterns of 10 test trials each is shown (two-sided Student’s t test). The thin dashed line indicates the level of chance (from 10 patterns and 10 onsets: 1%). (F) The model network is fed with a stimulus whose firing rate envelope is of varying maximum amplitudes stimulus ${\text{δν}}_{\text{a}}^{\text{max}}$ (amplitude values are color coded). (G) Mean and standard deviations over n = 10 trials of the increase in excitatory population activity ${\text{δν}}_{\text{e}}\left(\text{t}\right)={\text{ν}}_{\text{e}}\left(\text{t}\right)-{\text{ν}}_{\text{e}}^{\text{stat}}$ in the AD regime. In the inset, the response average in the time window T (highlighted by a gray bar along the time axis) as a function of the maximum amplitude of the stimulus ${\text{δν}}_{\text{a}}^{\text{max}}$ is shown. Note that the slope in the log-log input-output curves as a function of $\nu_{a}$ (lower insets in B and C) is not directly informative about the linear gain because of the different ranges in $\nu_{a}$ and $\nu_{e}$ (see Figure 4B). (H) Same as in (F) but for the RD regime. (I) Slope of the relationship between ${\langle {\text{δν}}_{\text{e}}\rangle }_{\text{T}}$ and ${\text{δν}}_{\text{a}}^{\text{max}}$ (mean ± SEM over n = 10 trials; statistical analysis: two-sided Student’s t test). (J) Decoding the sub-network rate waveform with a nearest-neighbor classifier. The thin dashed line indicates the level of chance (from the five waveforms shown in F: 20%). The mean accuracy ± SEM over five patterns of 10 test trials each is shown (two-sided Student’s t test). See also Figure S9.

Does the shift between activity states within the spectrum affect the capabilities of the circuit to encode afferent information? To address this question, we designed two types of afferent stimulus sets that we fed to the model, both in the AD regime and in the RD regime (Figure 7).

The first stimulus set mimicked the precise spatiotemporal patterns often evoked by sensory stimuli (Foffani et al., 2009, Luczak et al., 2015, Panzeri et al., 2010, Petersen et al., 2008, Urbain et al., 2015). It consisted of a pattern of sequential presynaptic co-activations, distributed over 500 ms, and it targeted a subset of 100 neurons within the network (see STAR Methods). We show in Figure 7A an example of such an afferent pattern. Figures 7B and 7C show the response in the targeted sub-network over different trials for the AD and RD regimes, respectively. The network activity across trials was highly structured by the stimulus in the AD regime (Figure 7B), while the stimulus-evoked response was less reliable in the RD regime (Figure 7C). We generated various random realizations of such afferent patterns (see Figure S9A) and analyzed the reliability of the responses across trials using a scalar metric for MUA (van Rossum 2001; see STAR Methods). We found that the trial-to-trial cross correlation between the output spiking responses and the presented afferent pattern was significantly larger in the AD than in the RD regime (p = 6e−3, paired t test; see Figure 7D). By including the distance of the above metric in a nearest-neighbor classifier, we constructed a decoder retrieving both the pattern identity and the stimulus onset from the output spiking activity of the target population (see STAR Methods). We used this classifier to analyze whether the ability of the AD regime to generate reliable output patterns (reported in Figure 7D) would lead to a robust joint decoding of both the identity and onset timing of the afferent input pattern. We found that these spatiotemporal features of the afferent input pattern were faithfully encoded by the activity of the target network in the AD regime (accuracy for the joint decoding of both afferent pattern identity and onset: 83.0% ± 10.1%; Figure 7E). In contrast, in the RD regime the decoding accuracy remained close to the level of chance (7.0% ± 9.0%; Figure 7E). The explanation for this difference can be found in the drastically different levels of activity in the two regimes ($\nu_{e}$ = 0.02± 0.01 Hz for the AD regime compared to $\nu_{e}$ = 25.9 ± 0.6 Hz for RD). In the AD regime, the patterned structure of the input strongly constrained the spiking activity of the population (as the stimulus-evoked spikes represented 92.4% ± 4.4% of the overall activity), therefore leading to a reliable encoding of the input identity. In the RD regime, the stimulus-evoked spiking was confounded by the strong ongoing dynamics in single trials in the RD regime (stimulus-evoked activity only represented 6.7% ± 5.7% of the overall activity) therefore impeding a reliable decoding of activity patterns.

We then fed the network with waveforms of afferent activity at various amplitudes, targeting the entire network without any spatiotemporal structure within the stationary period of the afferent waveform (see STAR Methods and Figure 7F). We decoded the level of afferent activity from the sum of the excitatory population activity within the recurrent network (Figures 7G and 7H, for the AD and RD regimes, respectively). In the RD regime, the network showed a linear response of high gain (Figure 7I and inset of Figure 7H) and the response waveforms accurately represented the level of the afferent input (see Figure 7H) (Murphy and Miller, 2009, Tsodyks and Sejnowski, 1995, van Vreeswijk and Sompolinsky, 1996). We found that, however, this was not the case in the AD regime. In this regime, the population response exhibited a weak amplification of the input signal (Figure 7I) and it failed to accurately follow the input (single trial responses in the AD regime had significantly lower cross correlations with the input waveform: 0.81±0.26 for AD versus 0.87±0.23 for RD, p = 4.4e−3, two-tailed Student’s t test). When decoding the input signal from the single-trial time-varying rate of a small populations of the network (100 excitatory neurons), we observed a higher decoding accuracy in the RD regime than in the AD regime (Figure 7J; see STAR Methods), suggesting that the RD regime favors the reliable encoding of the overall strength of the afferent activity thanks to its high amplification properties.

## Discussion

Our study reports an emergent feature of recurrent dynamics in spiking network models: a spectrum of asynchronous activity states in which firing activity spans orders of magnitude and in which the predominance of the synaptic activity shifts from the AD to the RD. Importantly, the continuous set of network states predicted by the model matches the set of non-rhythmic cortical states observed in awake rodents. Moreover, we found that, under specific biophysical constraints (discussed below), two different computational properties could coexist within the same network: the reliable encoding of complex presynaptic activity patterns in the AD regime together with the fast and high-gain response properties associated with the RD regime.

Using rate-based models, previous work suggested that recurrent networks can be made to operate in afferent-driven regimes and recurrent-driven regimes (Ahmadian et al., 2013, Rubin et al., 2015). However, this seminal work could neither investigate the detailed biophysical mechanisms behind the creation and coexistence of these regimes in the same network nor fully reveal the computational advantages in terms of information coding of each state resulting from their spiking dynamics. The present study developed those aspects through the combination of network modeling and experimental recordings in awake rodents.

### Key Features of the Model Necessary for the Emergence of the Spectrum

Unlike previous analysis where afferent synaptic currents were described by stochastic processes only constrained by a mean and a variance (Brunel, 2000, Renart et al., 2004, van Vreeswijk and Sompolinsky, 1996), we explicitly modeled afferent activity as a shot noise process producing post-synaptic events of excitatory currents. At the single-cell level, this feature was crucial to producing a skewed membrane potential distribution (DeWeese and Zador, 2006, Richardson and Swarbrick, 2010, Tan et al., 2014). At the network level, this enabled the emergence of the AD regime. Crucial to our model was the presence of conductance-based interactions. This feature of the model allowed synaptic efficacy to be high at low levels of activity while being strongly dampened at higher level (Kuhn et al., 2004). This property constrained an uncontrolled increase of the $V_{m}$ fluctuations upon a two to three orders of magnitude raise in recurrent activity and helped in maintaining stable asynchronous dynamics over the large range of ${\text{ν}}_{\text{e}}$. This feature of single-cell integration is not a sufficient condition and the non-monotonic $\sigma_{V}\;$-$\mu_{V}\;$ relationship is not generally observed in the network model (Figures 2 and 3).

The key variable governing network state modulation in the model was the level of afferent excitation. In agreement with such a dependence, shifts in the network state in the cortex can be controlled by thalamic excitation (Poulet et al., 2012). Network state modulation has also been shown to be regulated by the activity of other subcortical structures (Reimer et al., 2016, Zagha and McCormick, 2014). However, it remains to be established whether the contribution of subcortical structures is only mediated by a net increase in afferent excitatory input (Figure 1), by the neuromodulation of effective synaptic weights (Figure 2), by a combined effect of such modulations, or by other mechanisms.

Another network setting critical to obtaining the spectrum of regimes was the moderate strength of recurrent interactions (Figure 2). Whether this condition is met experimentally is difficult to assess, given the high heterogeneity of excitatory and inhibitory cells found in the neocortex, and given the area, layer, and species specificities that are often experimentally observed. This complexity notwithstanding, we restrict our discussion here to mouse experimental data on cortical layer 2/3. Unitary post-synaptic potentials observed in slice recordings (maximum amplitudes below 2 mV [Jiang et al., 2015, Lefort et al., 2009, Markram et al., 2015]) are compatible with the “moderate weights” that we used for both excitatory and inhibitory synaptic transmission (at −70 mV, our model gives maximal amplitudes of δV = 2.1 mV for excitatory synapses and δV = −1.4 mV for inhibitory synapses; Figure 1A). Moreover, from local measurements of excitatory projections in an adult rodent cortex (Seeman et al., 2018), recurrent excitatory connections seem to match the “sparse connectivity” requirement with connectivity probabilities below 10% (slightly higher values in the 10%–20% range were observed in juvenile animals [Lefort et al., 2009, Markram et al., 2015]). In contrast, local measurements of inhibitory projections in adult mice show high (> 30%) connectivity probabilities (Jiang et al., 2015; see also Barth et al., 2016). However, connectivity values of inhibitory projections largely vary depending on the type of source and target neurons (Pfeffer et al., 2013). This high heterogeneity across interneuronal subtypes may thus result in moderate connectivity values after averaging over inhibitory projections, despite some interneuronal classes showing high connectivity with specific targets. Altogether, although previous experimental observations provide evidence in support of our model setting, the extent to which moderate strength of recurrent interactions in the neocortex can be extended across different cortices and animal species remains to be determined. This is even more true considering that the strength of recurrent connectivity may vary over time (e.g., at different developmental stages and in an activity-based manner).

### Electrophysiological Properties of Non-rhythmic Network States: Model versus Experiments

Although we presented theoretical analyses of the dependence of the network dynamics on the afferent firing rate ${\text{ν}}_{\text{a}}$, we decided to compare real data and the model in evaluating how ${\text{ν}}_{\text{e}}$ and several higher-order membrane potential properties of cortical neurons depend on $\mu_{V}\;$, rather than on ${\text{ν}}_{\text{a}}$. The model prediction of the relationship between $\mu_{V}\;$ and ${\text{ν}}_{\text{e}}$ and $\mu_{V}\;$ and higher-order membrane potential properties was computed by combining two different model predictions (Figure 3): the dependence of ${\text{ν}}_{\text{e}}$ and higher-order membrane potential properties on ${\text{ν}}_{\text{a}}$, and the dependence between ${\text{ν}}_{\text{a}}$ and $\mu_{V}\;$. One caveat arising from such an approach is that it does not allow a direct verification that both such model features hold in real data. Although this caveat is partly alleviated by the fact that we propose to compare multiple relationships computed from the model and measured from the data, verification of both relationships would require measuring the ${\text{ν}}_{\text{a}}$ to the cortical network (S1) while it undergoes transitions in network states. However, fully monitoring the level of afferent input to S1 (or any other cortical network) at the experimental level is hard to achieve, because it would require monitoring the activity of all afferent populations, as well as of all neuromodulatory factors. The experimental measure of the relationship between ${\text{μ}}_{\text{V}}$ and ${\text{ν}}_{\text{e}}$ in real data remains however of significant interest for the following reasons. First, the experimentally measured membrane potential integrates the effect of multiple sources of afferent activity. Second, given that we showed that the relationship between ${\text{μ}}_{\text{V}}$ and ${\text{ν}}_{\text{e}}$ depends critically on the parameters and dynamic regimes of the considered network (Figure 3), this relationship is informative to understanding network dynamics.

The prediction that ${\text{ν}}_{\text{e}}$ spans three orders of magnitude as a function of $\mu_{V}\;$ (Figure 3) may contrast with previous studies reporting a much smaller range of firing rate variation during wakefulness (Watson et al., 2016, Hengen et al., 2016). However, those studies analyzed network dynamics at the time scale of homeostatic regulations (between 15 and 20 min) and slower temporal scales are expected to average away the faster, seconds-scale dynamics investigated in our study (Figure S7).

### Hypothetical Functions of Non-rhythmic Waking States

The transition toward aroused states elicits desynchronization of network activity (Harris and Thiele, 2011). This is thought to facilitate sensory processing through an increase in the signal-to-noise ratio of sensory-evoked activity (Busse et al., 2017, Harris and Thiele, 2011). However, the functional modulation of sensation within the various non-rhythmic substates of wakefulness remains unknown. Our model suggests that neocortical networks can switch their encoding mode upon changes of the afferent excitatory input, to either faithfully encode complex patterns of presynaptic activity (in the AD regime) or to exhibit strong population-wide recurrent amplification of the level of afferent input (in the RD regime). Experimentally, the behavioral state of the animal, indexed based on pupil size and running speed into low arousal, moderate arousal, and hyper arousal, was shown to modulate the $V_{m}$ signature of cortical dynamics similarly to what was observed in the model (McGinley et al., 2015b, Reimer et al., 2014, Busse et al., 2017). Importantly, under this definition of arousal state, arousal levels vary frequently and rapidly in head-fixed awake mice, with each arousal state lasting a few seconds and transitions between states happening within a few hundreds of milliseconds, in agreement with the time scales analyzed in this work. Comparing the experimental data presented in those studies with the predictions of our study, the AD regime could correspond to the moderate arousal state while the RD regime could correspond to the hyper arousal regime. Interestingly, moderate arousal was found to be optimal for the discrimination of a tone-in-noise auditory stimulus (McGinley et al., 2015b), a result in agreement with our model’s prediction of more reliable assembly activation in the AD regime (Figure 7D). In contrast, during locomotion (hyper arousal) neuronal responses in the visual system were found to be enhanced at all stimulus orientations (Reimer et al., 2014), consistent with the prediction of an unspecific recurrent amplification of population activity in the RD regime (Figure 7H).

Because the precise spatiotemporal pattern of neural responses within sensory cortices is thought to encode stimulus identity (Luczak et al., 2015, Panzeri et al., 2010), the AD regime might be an activity regime optimized for sensory discrimination. In contrast, the fast and unstructured amplification of excitatory inputs that characterizes the RD regime may potentiate the cortical response to weak sensory stimuli and could therefore represent a regime optimized for sensory detection. Future work focusing on the modulation of sensation in awake-behaving animals across various sensory modalities will test the validity and generality of this theoretical framework.

## STAR★Methods

### Key Resources Table

**Table:** 

REAGENT or RESOURCE	SOURCE	IDENTIFIER
**Experimental Models: Organisms/Strains**		

C57BL/6J mice	Charles River, Calco, Italy	Stock #:000027
B6;129P2-^Pvalbtm1(cre)Arbr/J^ mice	Jackson Laboratory, Bar Harbor, USA	Stock #:008069

**Software and Algorithms**		

Brian2	https://brian2.readthedocs.io	RRID:SCR_002998
Scipy	https://scipy.org	RRID:SCR_008058
Scikit-learn	https://scikit-learn.org/	RRID:SCR_002577
Neo	https://neo.readthedocs.io	RRID:SCR_000634
pClamp	Molecular Devices	RRID:SCR_011323

### Contact for Reagent and Resource Sharing

Further information and requests for resources and reagents should be directed to and will be fulfilled by the Lead Contact, Tommaso Fellin (tommaso.fellin@iit.it).

### Experimental Model and Subject Details

Experimental procedures involving animals have been approved by the IIT Animal Welfare Body and by the Italian Ministry of Health (authorization # 34/2015-PR and 125/2012-B), in accordance with the National legislation (D.Lgs. 26/2014) and the European legislation (European Directive 2010/63/EU). Experiments were performed on young-adult (4-6 weeks old, either sex) C57BL/6J n = 4 mice (Charles River, Calco, Italy) and PV-IRES-Cre n = 4 mice (B6;129P2-^Pvalbtm1(cre)Arbr/J^, Jackson Laboratory, Bar Harbor, USA). The animals were housed in a 12:12 hr light-dark cycle in singularly ventilated cages, with access to food and water *ad libitum*.

### Method Details

#### *In vivo* electrophysiology in awake mice

The experimental procedures for *in vivo* electrophysiological recordings in awake head-fixed mice have been previously described (Zucca et al., 2017). Briefly, a custom metal plate was fixed on the skull of young (P22-P24) mice two weeks before the experimental sessions. After a 2-3 days recovery period, mice were habituated to sit quietly on the experimental setup for at least 7-10 days (one session per day and gradually increasing session duration). The day of the experiment, mice were anesthetized with 2.5% isofluorane and a small craniotomy (0.5 mm x 0.5 mm) was opened over the somatosensory cortex and a 30 minutes long recovery period was provided to the animal before starting the recordings. Brain surface was kept moist with a HEPES-buffered artificial cerebrospinal fluid (ACSF). Current-clamp patch-clamp recordings were carried out on superficial pyramidal neurons (100 – 350 μm). 3–6 MΩ borosilicate glass pipettes (Hilgenberg, Malsfeld, Germany) were filled with an internal solution containing (in mM): K-gluconate 140, MgCl_2_ 1, NaCl 8, Na_2_ATP 2, Na_3_GTP 0.5, HEPES 10, Tris-phosphocreatine 10 to pH 7.2 with KOH. For simultaneous recordings of multi-unit activity (Figure S8), an additional glass pipette filled with ACSF was lowered into the tissue with the deeper tip placed at ∼300 μm from pial surface. Electrical signals were acquired using a Multiclamp 700B amplifier, filtered at 10 kHz, digitized at 50 kHz with a Digidata 1440 and stored with pClamp 10 (Molecular Devices, San Jose, USA). We recorded from n = 14 cells in N = 4 Wild-Type (WT) C57BL/6J animals. In the analysis, we added data from n = 8 cells in N = 4 PV-Cre mice obtained in recordings that were designed for a previous publication (Zucca et al., 2017). Those recordings contained period of optogenetic stimulation (every 5 s, see details in Zucca et al., 2017) of PV cells intermingled with period of spontaneous activity. The stimulation epochs and subsequent 500 ms-long time periods were discarded from the analysis in the additional 8 cells of PV-Cre mice. All the relations displayed in Figure 5 for the pooled data (WT + PV-Cre) were found similarly significant in the dataset containing only the WT mice (p < 1e-3 for all relations with similar correlation coefficients, see Figure S6C).

#### Computing the electrophysiological properties of non-rhythmic epochs

From the previously described recordings, we extracted stable membrane potential samples. Cells or periods with action potential peaking below 0mV or displaying a slow (∼1min) drift in the $V_{m}$ trace were discarded from the analysis. This resulted in dataset of n = 22 cells with a recording time per cell of 5.1±3.2 min. This stability criterion enabled us to perform the analysis on an absolute scale of membrane potential values (see Figures 5 and S6).

We first estimated a time-varying low frequency power within the $V_{m}$ samples: $Pow_{\left[2,10\right]Hz}^{max}\left(t\right)$. To this purpose, we discretized the time axis over windows of 500ms sliding with 25ms shifts and extracted the maximum power within the [2,10]Hz band (estimated with a fast Fourier transform algorithm, numpy.fft). All segments whose center $t_{i}$ had a $Pow_{\left[2,10\right]Hz}^{max}\left(t_{i}\right)$ value greater than the *rhythmicity threshold* were classified as “rhythmic” and discarded from future analysis. The value of the *rhythmicity threshold* was adjusted so that 50% of the data should be classified as “rhythmic” (see Figure 5C, in Figure S6D we analyze various *rhythmicity threshold* levels). In the remaining “non-rhythmic” samples ${\left\{t_{i}\right\}}_{NR}$, we evaluate the mean depolarization level $\mu_{V}\left(t_{i}\right)$ over the same 500ms interval surrounding the center time $t_{i}$ (T = 500ms is a good tradeoff between an interval short enough to catch the potential variability in network regimes at the sub-second timescale, i.e., T < 1 s, and an interval long enough to overcome the relaxation time of the network dynamics, i.e., T≫10ms, see main text). At that point, each time $t_{i}$ is associated to a given depolarization level $\mu_{V}\left(t_{i}\right)$. We now discretize the $\mu_{V}\;$ axis in j∈[1,20] points from −80mV to −50mV and we count the number of segments $n_{j}$ over all $t_{i}$ where $\mu_{V}\left(t_{i}\right)\in \left[\mu_{V}^{j},\;\mu_{V}^{j+1}\right]$ (see Figure S6A). As all cells did not contribute equally to all $\mu_{V}\;$ levels (see Figure S6C), we applied a “minimum contribution” criteria: if a depolarization level counted less than 200 segments ($n_{j}$ < 200), the $\left[\mu_{V}^{j},\;\mu_{V}^{j+1}\right]$ level was discarded from future analysis (see Figure S6A). We then count the number of spikes falling in a given level $\left[\mu_{V}^{j},\;\mu_{V}^{j+1}\right]$ level by counting spikes within the 500ms window. Spikes were detected as a positive crossing of the −30mV level (spikes were blanked in the $V_{m}$ traces by discarding the values above this threshold). We then computed the fluctuations properties of all depolarization levels. This was achieved by constructing a “pooled distribution” and a “pooled autocorrelation function” corresponding to all $\left[\mu_{V}^{j},\;\mu_{V}^{j+1}\right]$ intervals. For all $\left[\mu_{V}^{j},\;\mu_{V}^{j+1}\right]$ intervals, we took 500ms samples around all $t_{i}$ matching $\mu_{V}\left(t_{i}\right)\in \left[\mu_{V}^{j},\;\mu_{V}^{j+1}\right]$ and incremented the “pooled distribution” with those $V_{m}$ samples. Similarly, we incremented the “pooled autocorrelation function” with the individual normalized autocorrelation functions (evaluated up to 100ms time shift) of those $V_{m}$ samples. The resulting “pooled distributions” and “pooled autocorrelation functions” are illustrated for a single cell on Figure S6A. The “pooled distributions” at all $\left[\mu_{V}^{j},\;\mu_{V}^{j+1}\right]$ levels were used to evaluate the standard deviation $\sigma_{V}^{j}$ and skewness $\gamma_{V}^{j}$ while the “pooled autocorrelation functions” were used to determine the *autocorrelation time* $\tau_{V}^{j}$. The *autocorrelation time* $\tau_{V}^{j}$was determined by a numerical integration of this normalized autocorrelation function (Zerlaut et al., 2016). This procedure was repeated for all cells (shown in Figure S6B) and yielded the population data of Figure 5. We also analyzed the goodness-to-fit of a Gaussian fitting of the “pooled distributions,” we performed a least-square fitting (using the function scipy.optimize.leastsq) and we report the coefficient of determination $R^{2}\;$ (see main text).

#### Numerical simulations of recurrent network dynamics

We studied two versions of recurrently connected networks targeted by an afferent excitatory population: 1) a model with two coupled populations (excitatory and inhibitory neurons) and 2) a three population model with excitatory, inhibitory and disinhibitory neurons. Single cells were described as single compartment Integrate and Fire models with conductance-based exponential synapses. Their membrane potential dynamics thus follows the set of equations:

(1) $$\{\begin{array}{c} C_{m}\frac{dV}{dt}=\;g_{L}\;\left(E_{L}-V\right)+G_{e}\left(t\right)\left(E_{e}-V\right)+G_{i}\left(\;t\right)\left(E_{i}-V\right)\\ G_{e}\left(t\right)=\;\sum_{\left\{t_{e}\right\}}Q_{e}\;e^{-\frac{t-t_{e}}{\tau_{e}}}H\left(t-t_{e}\right)+\sum_{\left\{t_{a}\right\}}Q_{a}\;\;e^{-\frac{t-t_{a}}{\tau_{a}}}H\left(t-t_{a}\right)\\ G_{i}\left(t\right)=\;\sum_{\left\{t_{i}\right\}}Q_{i}\;e^{-\frac{t-t_{i}}{\tau_{i}}}H\left(t-t_{i}\right)+\sum_{\left\{t_{d}\right\}}Q_{d}\;e^{-\frac{t-t_{d}}{\tau_{d}}}H\left(t-t_{d}\right) \end{array}$$

Where H is the Heaviside (step) function. Note that, to emphasize the similarity in the equation between the different cell types considered (excitatory, inhibitory and disinhibitory), we omitted the index of the target cell (e.g., the weight should be $Q_{ae}$ for the afferent excitation onto the excitatory cell instead of $Q_{a}$ here). This set of equation is complemented with a threshold and reset mechanism, i.e., when the membrane potential V reaches a threshold $V_{thre}$ it is reset at the value $V_{reset}$ during a refractory period $\tau_{refrac}$. The sets of events $\left\{t_{X}\right\}$ corresponds to the synaptic events targeting a specific neuron. All parameters can be found on Table S1 for the two population model (Figure 1). The additional parameters required for the coupled three population model (excitation, inhibition, disinhibition, for Figures 4 and 7) can be found on Table S2.

Recurrent connections were drawn randomly by connecting each neuron of the population Y with $p_{XY}N_{X}$ neurons of the population X. Afferent drive of frequency $\nu_{a}$ onto population X with connectivity probability $p_{aX}$ was modeled by stimulating each neuron of the population X with a Poisson process of frequency $p_{aX}N_{a}\nu_{a}$ (i.e., using the properties of Poisson processes under the hypothesis of independent processes).

Numerical simulations were performed with the Brian2 simulator (Goodman and Brette, 2009). A time step of dt = 0.1ms was chosen. Stationary properties of network activity were evaluated with simulations lasting 10 s. The first 200ms were discarded from the analysis to remove the contributions of initial transients. Simulations were repeated over multiple seeds generating different realizations of the random connectivity scheme and of the random afferent stimulation (see number in the legends).

#### Mean field analysis of recurrent dynamics

We obtained an analytical estimate of the network activity in the numerical model by adapting the classical mean-field descriptions of network dynamics. In a nutshell (see Brunel and Hakim [1999] for further details and Renart et al. [2004] for review), the mean field approach provides a simplified, or reduced, description of the spike-based dynamics of the network in terms of the temporal evolution of the firing rates of the populations. To perform this reduction, we hypothesize that spike trains follow the statistics of Poisson point processes (and can therefore be statistically described by their underlying rate of events) and that all neurons receive an average synaptic inputs (the “mean-field*”*) derived from the mean connectivity property of the network and the firing rates of their input populations. From those hypotheses, it results that the firing rate of a population follows the behavior of a prototypical neuron whose dynamics is described by a simple equation relating its output firing rate to the set of rates of its input populations. For interconnected populations including recurrent connections, one therefore obtains a coupled dynamical system of a few variables (only the firing rates of the different populations considered) that can be analyzed and compared to the output of the numerical simulations (see main text and Figure S2). We describe in the following how we adapted such a theoretical description to capture the behavior of the network described in the main text.

For the set of rate equations describing population activity, we started from the first order of the Markovian formalism proposed in (El Boustani and Destexhe, 2009). For the two population model, the rates of the excitatory and inhibitory population ($\nu_{e}$, $\nu_{i}$ respectively) thus follow:

(2) $$\{\begin{array}{c} \frac{\partial \nu_{e}}{\partial t}=\;\frac{1}{T}\cdot \left(F_{e}\left(\nu_{e},\;\nu_{i},\;\nu_{a}\right)\;-\;\nu_{e}\right)\\ \frac{\partial \nu_{i}}{\partial t}=\;\frac{1}{T}\cdot \left(F_{i}\left(\nu_{e},\;\nu_{i},\;\nu_{a}\right)\;-\;\nu_{i}\right) \end{array}$$

Where T = 5ms arbitrarily sets the timescale of the Markovian description (not crucial here, as we limit our analysis to the stationary solution of this equation). Importantly, $\nu_{a}$ is not a variable of this system of equation as this is an external input. For simplicity we describe the theoretical framework for the two-population model only. A generalization to the three population model (see Figure 4A) is straightforward: one needs to introduce an equation describing the evolution of $\nu_{d}$ coupled to the $\nu_{i}$ term in Equation 2.

The functions $F_{e}$ and $F_{i}$ represent the *input-output* functions of the excitatory and inhibitory cells respectively: i.e., they relate the input firing rates to the output firing rate of each cell type given the cellular, synaptic and connectivity parameters (see Table S1). They constitute the core quantities of this theoretical framework. While more reductive biophysical models enable an analytical approximations for those *input-output* functions through stochastic calculus (reviewed in Renart et al., 2004), the situation considered here clearly impedes such analytical approach. Two reasons prevent this approach: 1) the previously mentioned analytical approach rely on the diffusion approximation (i.e., reducing the post-synaptic currents to a stochastic process of a given mean and variance) whereas some of the dynamics described here is led by higher-order fluctuations (typically, the strongly skewed distribution of excitatory currents is crucial for spiking in the sparse activity regime, see the main text) and 2) even in the fluctuation-driven regime where the diffusion approximation holds, the present model is too complex to be analyzed through the commonly used Fokker-Planck approach (we consider a model of conductance-based synapses with non-negligible synaptic dynamics). We therefore chose to adopt a semi-analytical approach (see Kumar et al., 2008 and Zerlaut et al., 2018) for a semi-analytical procedure similar to the one presented here): we simulated numerically the dynamics of single neuron dynamics at various stationary input rates $\left(\nu_{e},\;\nu_{i},\;\nu_{a}\right)$ and we calculated the output firing at each level for the two considered populations (excitatory and inhibitory), we thus obtain a numerical subsampling of the required F functions (see Figure S2A, note the important sampling of low activity levels). To convert this discrete sampling into an analytical function, we adapted a fitting procedure described previously (Zerlaut et al., 2016). Briefly, this previous study showed that a fitting of the output firing rate could be achieved by transforming the firing rate data into a *phenomenological threshold* where a linear fitting enables to obtain a stable and accurate minimization. We transposed this approach to capture the output firing probability both within-and-far from the diffusion approximation. This was achieved by adding higher order terms to the *phenomenological threshold*: 1) the skewness of the membrane potential distribution $\gamma_{V\;}$ and 2) the probability to be above threshold $P_{V>V_{thre}}$ given the third-order Edgeworth expansion of the membrane potential distribution (typically, two terms with a significant contribution in the sparse activity regime).

We present here the mathematical relations used to build up this procedure (all derivations were performed with the python module for symbolic computation: sympy and directly exported to numpy for numerical evaluation, see the associated Interactive notebook).

We start by calculating the properties of the subthreshold membrane potential fluctuations. Again, for simplicity, we omitted the index of the target population in the following notations. Adapting previous analysis (Kuhn et al., 2004, Zerlaut et al., 2016) to the shotnoise inputs (Equation 1), the expression for the mean $\mu_{V}\;$, standard deviation $\sigma_{V}\;$ and average autocorrelation time $\tau_{V}$ of the membrane potential fluctuations are given by:

(3) $$\mu_{V}=\frac{g_{L}\;E_{L}+\;\sum_{s\in \left\{e,\;i,\;a,\;d\right\}}p_{s}\;N_{s}\;\nu_{s}\;Q_{s}\;\tau_{s\;}E_{s}}{g_{L}+\;\;\sum_{s\in \left\{e,\;i,\;a,\;d\right\}}p_{s}\;N_{s}\;\nu_{s}\;Q_{s}\;\tau_{s\;}}$$

(4) $${\left(\sigma_{V}\right)}^{2}\;=\;\sum_{s\in \left\{e,\;i,\;a,\;d\right\}}p_{s}\;N_{s}\;\nu_{s}\frac{{\left(Q_{s}\tau_{s}\;\tau_{m}\left(E_{s}-\mu_{V}\right)/C_{m}\right)}^{2}\;\;}{2\;\left(\tau_{s}+\tau_{m}\right)}$$

(5) $$\tau_{V}=\frac{\;\sum_{s\in \left\{e,\;i,\;a,\;d\right\}}p_{s}\;N_{s}\;\nu_{s}{\left(Q_{s}\tau_{s}\frac{\tau_{m}\left(E_{s}-\mu_{V}\right)}{C_{m}}\right)}^{2}}{\sum_{s\in \left\{e,\;i,\;a,\;d\right\}}p_{s}\;N_{s}\;\nu_{s}/\left(\tau_{s}+\tau_{m}\right){\left(Q_{s}\tau_{s}\frac{\tau_{m}\left(E_{s}-\mu_{V}\right)}{C_{m}}\right)}^{2}}$$

Pushing the analysis of the shot noise to the third-order, one can also get the skewness of the distribution:

(6) $$\gamma_{V}=\frac{1}{\sigma_{V}^{3}}\sum_{s\in \left\{e,\;i,\;a,\;d\right\}}p_{s}\;N_{s}\;\nu_{s}\frac{{\left(Q_{s}\tau_{s}\;\tau_{m}\left(E_{s}-\mu_{V}\right)/C_{m}\right)}^{3}\;\;}{3\;\left(\tau_{s}+2\;\tau_{m}\right)\;\left(2\;\tau_{s}+\tau_{m}\right)}$$

From the three statistical moments of the $V_{m}$ distribution, one can get the third-order Edgeworth expansion of the membrane potential (Brigham and Destexhe, 2015):

$$P\left(V\right)=\frac{1}{\sqrt{2\pi }\;\sigma_{V}}\;e^{-\frac{{\left(V-\mu_{V}\right)}^{2}}{2\;\sigma_{V}^{2}}}\;\left(1+\frac{\gamma_{V}}{6}\;{\left(\frac{V-\mu_{V}}{\sqrt{2}\;\sigma_{V}}\right)}^{3}\right)$$

That we use to obtain a baseline estimate of the probability to be above threshold:

(7) $$P_{V>V_{thre}}=\int_{V_{thre}}^{\infty }P\left(V\right)dV=\sqrt{\frac{\pi }{2}}-\frac{1}{6}\left(\gamma_{V}-\frac{\gamma_{V}}{{\left(\sigma_{V}\right)}^{2}}{\left(V_{thre}-\mu_{V}\right)}^{2}+3\;\sqrt{2\;\pi }\;\;e^{\frac{{\left(V_{thre}-\mu_{V}\right)}^{2}}{2\;\sigma_{V}^{2}}}Erf\left(\frac{V_{thre}^{eff}-\;\mu_{V}}{\sqrt{2}\;\sigma_{V}}\right)\right)\;e^{-\frac{{\left(V_{thre}-\mu_{V}\right)}^{2}}{2\;\sigma_{V}^{2}}}\;$$

In this semi-analytical framework (Zerlaut et al., 2016), the formula linking the output firing rate $\nu_{out}$ and the *phenomenological threshold* $V_{thre}^{eff}\;$is:

(8) $$\nu_{out}=\frac{1}{2\;\tau_{V}}Erfc\left(\frac{V_{thre}^{eff}-\;\mu_{V}}{\sqrt{2}\;\sigma_{V}}\right)$$

Where Erfc is the complementary Error function (of inverse InvErfc). To determine the *phenomenological threshold* based on a set of observation of $\nu_{out}$ as a function of $\left(\nu_{e},\;\nu_{i},\;\nu_{a}\right)$, we translate $\left(\nu_{e},\;\nu_{i},\;\nu_{a}\right)$ into $\left(\mu_{V},\;\sigma_{V},\;\tau_{V},\;\gamma_{V},P_{V>V_{thre}}\right)$ and we invert the previous equation through:

(9) $$V_{thre}^{eff}=\;\mu_{V}+\;\sqrt{2}\;\sigma_{V}\;InvErfc\left(2\;\tau_{V}\;\nu_{out}\right)\;$$

and we fit a second-order polynomial of the form:

(10) $$V_{thre}^{eff}=p_{0}+\;\sum_{i\in \left[1,5\right]}p_{i}\;X_{i}+\;\sum_{\left(i,j\right)\in {\left[1,5\right]}^{2}}p_{ij}\;X_{i}\;X_{j}$$

where the $X_{i}$ terms are given by:

$$X_{1}=\frac{\mu_{V}-\;\mu_{V}^{0}}{\delta \mu_{V}^{0}},\;X_{2}=\frac{\sigma_{V}-\;\sigma_{V}^{0}}{\delta \sigma_{V}^{0}},\;X_{3}=\frac{\tau_{V}-\;\tau_{V}^{0}}{\delta \tau_{V}^{0}},\;X_{4}=\gamma_{V},\;X_{5}=P_{V>V_{thre}}$$

The normalization factors$\;\mu_{V}^{0}$ = −60mV, $\delta \mu_{V}^{0}$ = 10mV. $\sigma_{V}^{0}$ = 4mV, $\delta \sigma_{V}^{0}$ = 6mV, $\tau_{V}$ = 10ms and $\delta \tau_{V}^{0}$ = 20ms are arbitrary normalization constants (for the fitting, one needs to insure that all terms remain in the same order of magnitude: ∼[−1,1]). The linear fitting was performed by a linear least-squares minimization (Ridge regression) from scikit-learn (Pedregosa et al., 2011). We show on Figure S2A the result of this procedure: from the numerical sampling of the input-output function (dots with error bars in Figure S2A), the fitting enables to get an analytical function (plain lines in Figure S2A). We reproduce this procedure to obtain the two functions: $F_{e}$ and $F_{i}$ (shown in (i) and (ii) in Figure S2A, the fitting coefficients are reported on Table S3). We can now use Equation 2 to make theoretical predictions on the network activity as well as its signatures (in terms of membrane potential and synaptic currents in particular). Finding the stable fixed point was done by launching a trajectory ruled by the system Equation 2 starting from $\left(\nu_{e}^{0},\;\nu_{i}^{0}\;\right)$ = (0.02Hz, 0.02Hz). On Figure S2B we show the phase space of the dynamical system corresponding to Equation 2 and the trajectory that finds the fixed point of the dynamics $\left(\nu_{e}^{FP},\;\nu_{i}^{FP}\right)$ for the sparse activity state and the dense balanced state. On Figure S2C, we show how the stationary activity levels predicts the membrane potential signature of the two regimes by applying Equations 3, 4, 5, and 6.

#### Characterizing network dynamics

From the numerical simulations, we monitored all spike times and binned them in $T_{b}$ = 2ms time bins to obtain the spike train $S_{i}\left(t\right)$ for each neuron i ($S_{i}\left(t\right)$ takes only 0 or 1 values as $\tau_{refrac}>T_{b}$). We analyzed the network activity by looking at the time-varying firing rate of the population X:

$$\nu_{X}\left(t\right)=\frac{\sum_{i\;\in \;\left[0,N_{X}\right]}S_{i}\left(t\right)}{N_{X}}$$

We measured population synchrony by averaging the correlation coefficient of the spike trains over some (i,j) neuronal pairs (Kumar et al., 2008), i.e., the synchrony index SI was given by:

$$SI=\;{\langle \frac{Cov\left(S_{i},S_{j}\right)}{\sqrt{Var\left(S_{i}\right)Var\left(S_{j}\right)}}\rangle }_{i,j}$$

In practice we selected 4000 spiking neuronal pairs for numerical evaluation.

Additionally, we monitored the membrane potential, the synaptic conductances and the synaptic currents of four randomly chosen cells in each populations. To evaluate the mean, standard deviations, skewness and autocorrelation time of the membrane potential fluctuations, we discarded the refractory periods from the analysis. The same discarding procedure was applied for the mean conductances and currents reported here. The excitatory currents and conductances shown in the main text merge all excitatory contributions together (afferent and recurrent excitations). The inhibitory currents and conductances correspond to recurrent inhibition only for excitatory cells and add the disinhibitory contributions for inhibitory cells in the three population model.

#### Varying parameters of the network model

We investigated the robustness of the proposed theoretical picture by studying its sensitivity to parameter variations. The values of parameters and results of this analysis is shown on Figure S3. Network simulations were run with time step 0.1ms, lasted 10 s and were repeated over 4 different seeds.

#### Response to an afferent time-varying rate envelope

To emulate a time-varying afferent input onto the local cortical network (see Figure 4), we took an arbitrary waveform for the firing rate activity of the afferent population. From this waveform, an inhomogeneous Poisson process was generated to stimulate each neuron of the three populations model. For Figure 4, the waveform was taken as:

$$\nu_{a}\left(t\right)=\sum_{\{i\in \left[1,2,3\right]\}}A_{i}\left(1+Erfc\left(\frac{t-t_{i}}{T_{rise}}\right)\;\right)\left(1+Erfc\left(\frac{t_{i}+T_{length}-t}{T_{rise}}\right)\;\right)/4$$

with $A_{1}$ = 4Hz, $A_{2}$ = 18Hz, $A_{3}$ = 8Hz, $t_{1}$ = 100ms, $t_{2}$ = 1150ms,$\;t_{3}$ = 2000ms, $T_{rise}$ = 50ms and $T_{length}$ = 900ms. The resulting waveform is shown in Figure 4C.

#### Determining the relaxation time constant of the network dynamics

We determined the network time constant $\tau_{NTWK}$ at three different levels of network activity in the three-population model (see Figure 4F). The network model was stimulated with three different levels of stationary background activity $\nu_{a}$ = 4 Hz, $\nu_{a}$ = 8 Hz and $\nu_{a}$ = 18 Hz. On top of this background activity, we added a 2Hz step of afferent excitation lasting $T_{stim}$ = 100ms and each 500ms. We repeated this stimulation a 100 times and we computed the trial-average response to this stimulus (shown in the inset of Figure 4F). The network time constant was estimated by a least-square fitting of the following waveform: $\nu \left(t\right)=\nu_{e}^{0}+\delta \nu_{e}\;\left(\left(H\left(t\right)-H\left(t-T_{stim}\right)\right)\;\left(1-e^{-\frac{t}{\tau_{NTWK}}}\right)+H\left(t-T_{stim}\right)\;e^{-\frac{t-T_{stim}}{\tau_{NTWK}}}\right)$. The three values $\nu_{e}^{0}$, $\delta \nu_{e}$ and $\tau_{NTWK}$ were determined through the minimization procedure. We show the $\tau_{NTWK}$ values in the bar plot and the response amplitudes $\delta \nu_{e}$ as the scale bar annotations in Figure 4F.

#### Encoding of spiking patterns of presynaptic activity

We designed a stimulation to investigate whether a complex spatio-temporal pattern targeting a subset of the local cortical population could be faithfully encoded by the activity of this sub-network (see Figures 7A–7E). We took the following scheme. Within the 100 neurons of the afferent population, we made groups of 10 neurons that co-activate simultaneously. Those groups of 10 neurons target a subset of 100 neurons within the 4000 neurons of the excitatory population (with a synaptic weight equal to those of background afferent connections). Presynaptic neurons only make mono-synaptic connections to a target neuron, but two co-activated neurons may connect to the same neuron in the 100 neurons target population hence creating some degree of synchronous activation (but with a low probability: 1%, because $p_{ae}$ = 10%, see Table S1). Within a window of 500ms, we generate random activations over time with a homogeneous Poisson process of frequency 20 Hz (i.e., 10 activations per 500 ms window) and assign randomly each activation time to a given afferent group, this generates one pattern (see example patterns on Figures 7A and S9A). We reproduce this procedure 10 times with a different random seed to obtain 10 patterns of presynaptic activations. We then feed the network with this afferent pattern on top of the non-specific background afferent drive (both in the AD regime and in the RD regime). We run 20 trials per pattern, where the realization of the background activity varies while the pattern is kept constant. We compared the output spiking patterns using the inner-product (IR) and distance (D) for multi-neuron spike trains derived in (Houghton and Kreuz, 2012; Van Rossum, 2001) implemented in the publicly-available package pymuvr. This metrics takes a timescale τ that sets the temporal sensitivity (for τ→0 the metrics is only sensitive to infinitely precise coincident spiking, for τ→∞ the metrics is a joint spike count over time). The value of τ was set to 5 ms as this timescale was found in preliminary analyses to be the minimal timescale for which a reliable encoding of the input pattern was observed in the AD regime. The cross-correlation coefficient between spike trains computed in Figure 7D was computed as $CC\left(S_{1},\;S_{2}\right)=IR\left(S_{1},\;S_{2}\right)/\sqrt{IR\left(S_{1},\;S_{1}\right)\;IR\left(S_{2},\;S_{2}\right)}$, where IR is the inner product between two spike trains $S_{1}$ and $S_{2}$. We then implemented a k-neighbor classifier to decode the output spike train of the excitatory subnetwork. The distance between two output patterns relied on the distance D. We implemented this custom metrics in the k-neighbor-classifier of scikit-learn (Pedregosa et al., 2011) to obtain our classifier. We first train the classifier on the first 10 trials and tested on the last 10 trials per pattern. For a first-nearest-neighbor classification, we found the following decoding accuracies: 88.0 ±9.8% for AD activity and 16.0 ± 0.2% for RD activity. Raising the number of neighbors up to 10 points (over a training set containing 10 trials per presynaptic patterns) did not affect this difference: it yielded (non-monotonic) variations of the decoding accuracy between 90% and 65% for AD regimes and between 27% and 16% for RD regimes, we therefore kept a nearest-neighbor classifier for all analysis. To partially separate the spatial and temporal components in afferent patterns encoded by the network activity, we duplicated all 10 patterns and their 10 repetitions in the training set by aligning the network response onset time to all observed stimulus onsets (shifting the time axis, the procedure is depicted in Figure S9A). The final decoder should therefore associate a trial in the test set with a given pattern identity and a given stimulus onset (accuracy results shown in Figure 7E). In Figure S9B, we show the distributions of decoded stimulus onsets in the AD and RD regimes. The percentage of stimulus-evoked activity (see main text) was evaluated by comparing the firing rates in the 500 ms before and during the 500 ms of the stimulus.

#### Encoding of presynaptic rate waveforms

We designed a stimulation to investigate whether the rate envelope of given presynaptic stimulus could be faithfully encoded by the activity of the network (see Figures 7F–7J). The waveform was taken as:

$$\nu_{a}\left(t\right)=A_{bg}+A_{stim}\;\left(1+Erfc\left(\frac{t-t_{1}}{T_{1}}\right)\;\right)\left(1+Erfc\left(\frac{t_{2}-t}{T_{2}}\right)\right)/4$$

with $T_{1}$ = 100ms, $T_{2}$ = 300ms, $t_{1}$ = 400ms and $t_{2}$ = 1100ms. $A_{bg}$ = 4Hz to produce the AD regime and $A_{bg}$ = 14Hz to produce the RD regime. $A_{stim}$ was varied from 0.1Hz to 7Hz in 5 different levels (see the resulting waveforms are shown in Figure 7F). This time-varying rate was then converted to a Poisson process (varying the seed in all trials) setting the activity of the afferent population and fed as an input to the recurrent network. Similarly to the previous section, we implemented a k-neighbor classifier to decode the rate waveform from a sub-population of the network (taking the same 100 neurons sample). The time-varying rate of the subpopulation was computed by binning spikes in 2ms bins and Gaussian smoothing of extent 30ms, yielding the quantity R(t). The metric for the rate waveform decoder was the integral over the stimulus duration of the square difference between waveforms, i.e., for two waveforms R1 and R2, it corresponded to:

$$M\left(R1\left(t\right),\;R2\left(t\right)\right)=\;\int_{0}^{1s}{\left(R1\left(t\right)-\;R2\left(t\right)\right)}^{2}\;dt$$

We run 20 trials for each of the five levels of afferent activity shown in Figure 7F. We trained the decoder on the first 10 trials and tested it on the following 10 trials.

### Quantification and Statistical Analysis

Data were analyzed with SciPy (Oliphant, 2007). Experimental data were translated to the Python format using Neo (Garcia et al., 2014). In Figures 6A–6D, we performed least-square linear regressions on continuously distributed data (implemented in scipy.stats.linregress), we report the correlation coefficients (“c”). Given the partial temporal overlap between individual samples of membrane potential, the data across the different $\mu_{V}\;$ levels cannot be considered as independent, so we evaluated statistical significance (“p”) with a non-parametric one-tailed permutation test (performed with 1e5 permutations, hence p values were reported as “p<1e-5” if no permutation was found to exhibit the correlation value of the data). In Figure 7, we evaluated the significance of the difference in encoding accuracy and response gain with a two-sided t test (implemented in scipy.stats.ttest_rel).

### Data and Software Availability

The code for the numerical simulations and analysis producing the main and supplemental data is publicly available in the form of an *Interactive Python notebook* (Pérez and Granger, 2007) on the following link: https://github.com/yzerlaut/notebook_papers/blob/master/The_Spectrum_of_Asynch_Dynamics_2018.ipynb.

## Supplemental Information

Document S1. Figures S1–S9 and Tables S1–S3

Document S2. Article plus Supplemental Information
