# Supplementary material for: The Spectrum of Asynchronous Dynamics in Spiking Networks as a Model for the Diversity of Non-rhythmic Waking States in the Neocortex
Source: Cell Rep. 2019 Apr 23;27(4):1119–1132.e7. doi: 10.1016/j.celrep.2019.03.102 (PMC6486483; doi:10.1016/j.celrep.2019.03.102)
Supplement: Document S1. Figures S1–S9 and Tables S1–S3 [file mmc1.pdf]

Cell Reports, Volume *27*

Supplemental Information

The Spectrum of Asynchronous Dynamics in Spiking Networks as a Model for the Diversity of Non-rhythmic Waking States in the Neocortex

Yann Zerlaut, Stefano Zucca, Stefano Panzeri, and Tommaso Fellin

Supplementary Figures

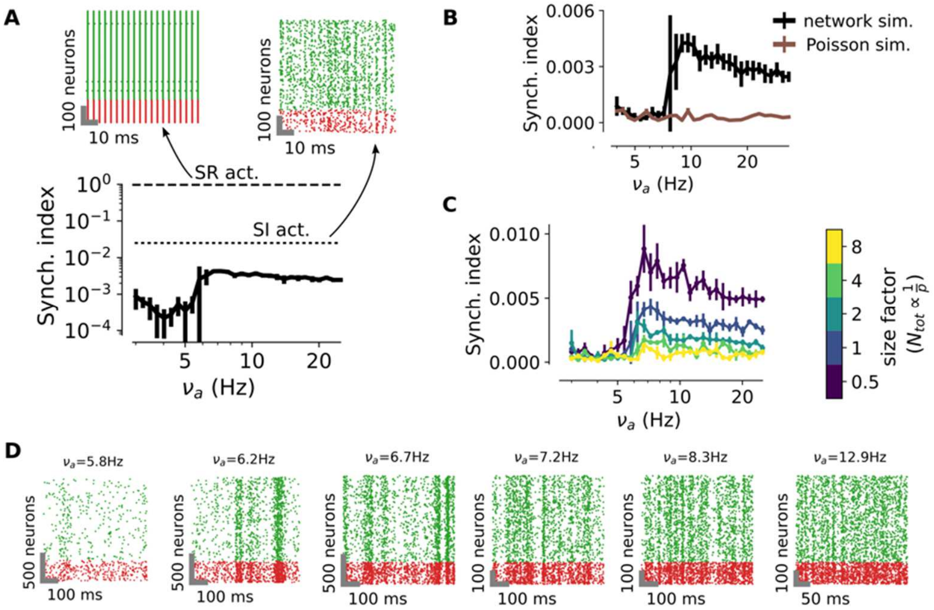

Figure S1. Finite size effects induce weakly synchronous fluctuations of population activity. Related to Figure 1. **(A)** The dynamics in a 5000 neurons network exhibit much lower synchrony than network configurations exhibiting either Synchronous Regular (SR) activity or Synchronous Irregular (SI) activity (Brunel, 2000). **(B)** However, the dynamics still exhibits significant residual synchrony compared to independent Poisson processes (Brown curve, generated according to the parameters and results of network simulations: sample time, temporal discretization and excitatory and inhibitory firing rates). **(C)** This residual synchrony is due to finite-size effects: the amount of residual synchrony is drastically reduced by increasing the number of neurons in the network (and concomitantly by decreasing the connectivity probability to keep the number of synapses equal, see the identical levels of excitation and inhibition at different sizes in **Figure S3H**). **(D)** Samples of activity showing transient synchronous activations at various levels of afferent inputs in a 5000 neurons network. Note that the synchronous activations in the moderate afferent activity range ($\nu_a \in [6,8]$ Hz, where the synchrony exhibits a maximum, see panel **C**) is due to transient stochastic transitions toward high activity regimes (i.e. neighboring levels of the spectrum). At higher activity levels the residual synchrony is classically due to short-lasting gamma-like patterns (Brunel and Hakim, 1999).

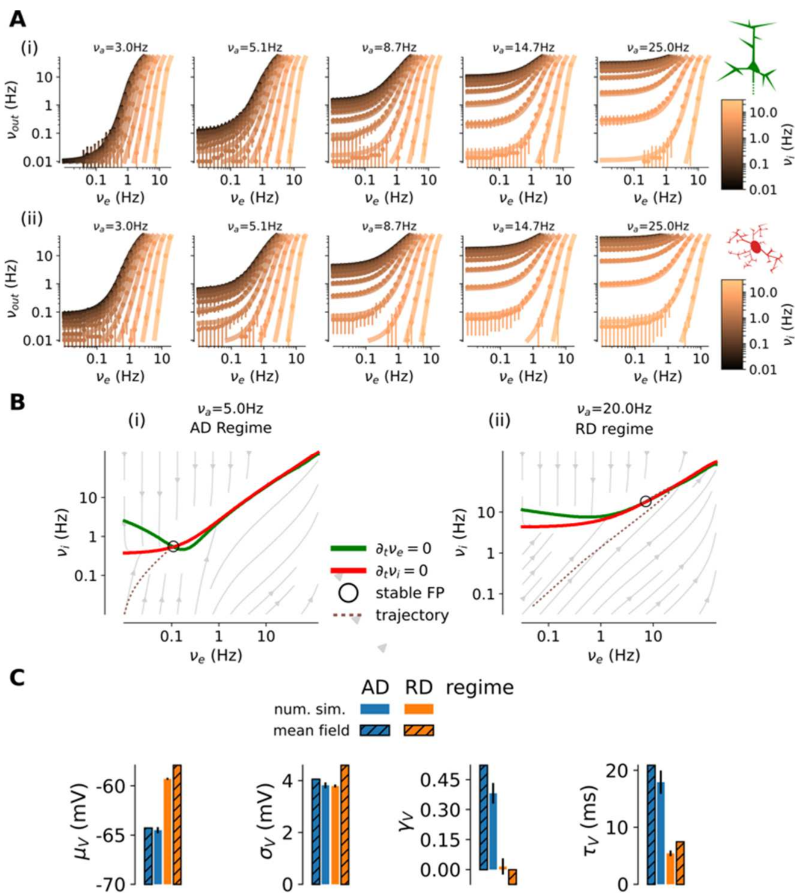

Figure S2. Theoretical analysis of network dynamics: semi-analytical derivation of the mean-field description and comparison with numerical simulations. Related to Figure 1. (A) Capturing the input-output functions of single cells through a semi-analytical approach (shown here for the excitatory cell in (i) and for the inhibitory cell in (ii)). We made numerical simulations of single neurons to have a numerical sampling of their input-output function. We varied (ν_e, ν_i, ν_a) and measured the output firing rate ν_{out} (ν_e is the x-axis, variations of ν_i are color coded and five of the ten levels of ν_a simulated are shown by the five plots from left to right). This numerical sampling (dots and error bars) is fitted by an analytical function (transparent plain lines), see **"Mean Field Analysis"** section. **(B)** Dynamical system analysis to find the stable fixed point of the dynamics. We show the phase space of the dynamical system with its vector field. We show the trajectory (brown line) used to find the fixed point for the AD regime (i) and the RD regime (ii), i.e. ν_a = 5 Hz and ν_a= 20 Hz respectively). Those theoretical firing levels (computed for all ν_a levels) are shown on **Figure 1B** and were used to compute the predictions of the synaptic current ratio I_e^{aff}/I_e and $|I_i/I_e|$ shown in **Figure 1C**. **(C)** Comparison between theoretical predictions and numerical simulations for the membrane potential signature in the AD regime and RD regime.

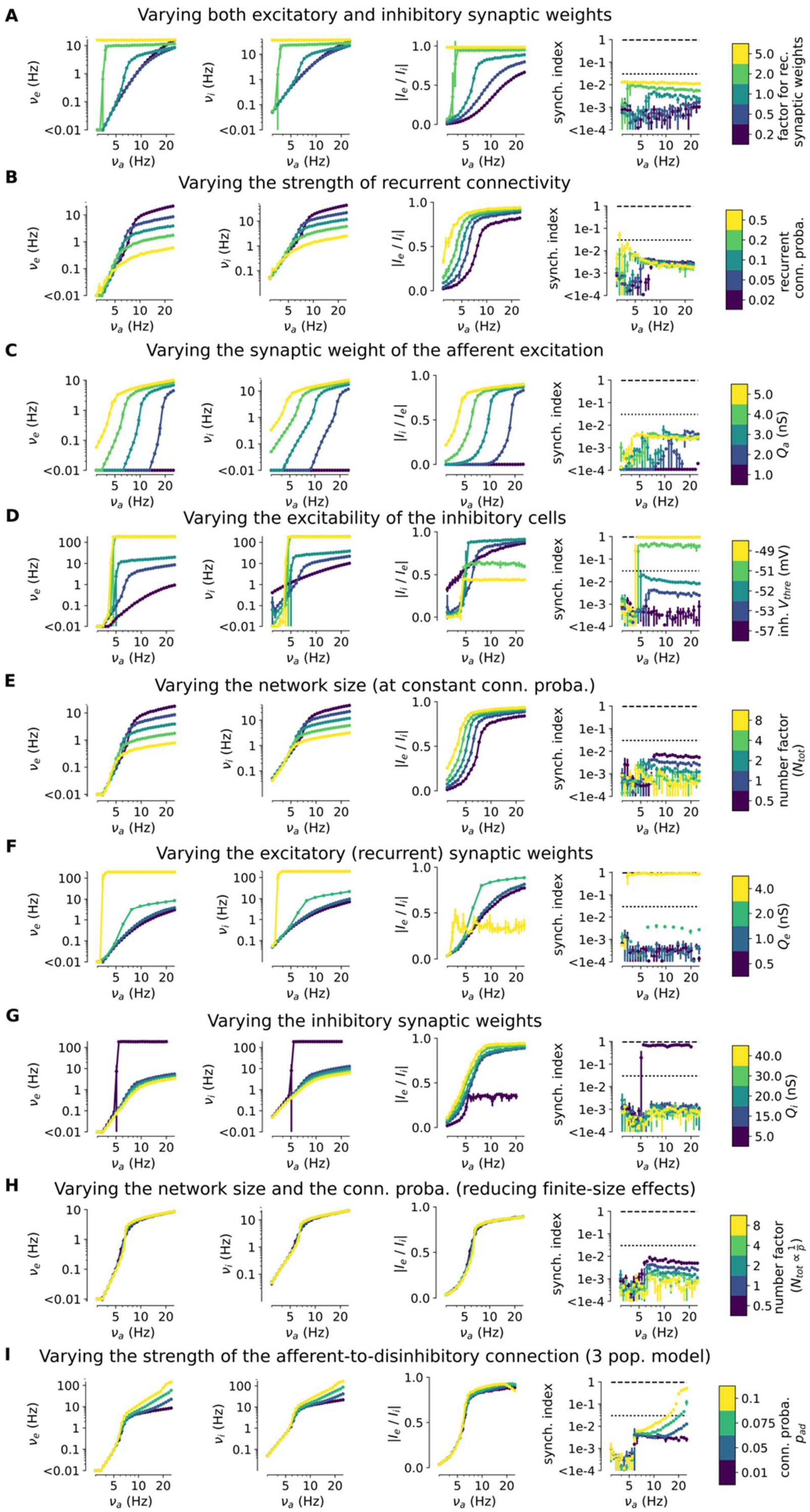
A
Varying both excitatory and inhibitory synaptic weights
factor for rec. synaptic weights
5.0
2.0
1.0
0.5
0.2
B
Varying the strength of recurrent connectivity
recurrent conn. proba.
0.5
0.2
0.1
0.05
0.02
C
Varying the synaptic weight of the afferent excitation
Q_a (nS)
5.0
4.0
3.0
2.0
1.0
D
Varying the excitability of the inhibitory cells
inh. V_{thre} (mV)
-49
-51
-52
-53
-57
E
Varying the network size (at constant conn. proba.)
number factor (N_{tot})
8
4
2
1
0.5
F
Varying the excitatory (recurrent) synaptic weights
Q_e (nS)
4.0
2.0
1.0
0.5
G
Varying the inhibitory synaptic weights
Q_i (nS)
40.0
30.0
20.0
15.0
5.0
H
Varying the network size and the conn. proba. (reducing finite-size effects)
number factor ($N_{tot} \propto \frac{1}{p}$)
8
4
2
1
0.5
I
Varying the strength of the afferent-to-disinhibitory connection (3 pop. model)
conn. proba. p_{ad}
0.1
0.075
0.05
0.01
ν_e (Hz)
ν_i (Hz)
$|I_e / I_i|$
$|I_i / I_e|$
synch. index
ν_a (Hz)

Figure S3. Emergence of the spectrum of asynchronous dynamics: robustness against parameter variations in the network model. The presence of the spectrum is here quantified by variations of the spiking activity (ν_e and ν_i) over 2-3 orders of magnitude and a smooth $|I_i/I_e|$ curve ranging from excitatory-dominated dynamics $|I_i/I_e| \sim 0$ to a balanced setting $|I_i/I_e| \sim 1$ upon variations of the afferent activity level. Related to Figure 2. **(A)** Varying the strength of excitatory and inhibitory recurrent synaptic weights (subsample of the data shown in main text). Here the dynamics remains asynchronous (see the low synchrony index) but the spectrum disappears, at high synaptic weight factors the dynamics only displays balanced activity, i.e. $|I_i/I_e| \sim 1$. **(B)** Varying the strength of recurrent connectivity (i.e. raising the number of both excitatory and inhibitory synapses while keeping the total number of neurons constant). Here, while the spectrum is still present for low connectivity probabilities $p < 0.2$, the spectrum tends to disappear when the network is densely connected $p \geq 0.5$, in accordance with the need of low strength of recurrent interactions (see main text). Note also the strong decrease in firing rates when raising the connectivity as consequence of an architecture (connectivity and synaptic weights) dominated by inhibition. **(C)** Varying the weight of afferent excitation (both on excitation and inhibition i.e. Q_{ae} and Q_{ai}). We see that the value of the afferent weight sets the range of afferent activity (ν_a) where the spectrum appears. The spectrum of asynchronous dynamics is nonetheless present within this range of synaptic weights (see the various levels of $|I_i/I_e|$ and the low synchrony index of the activity). **(D)** Varying the excitability of the inhibitory cells. **(E)** Varying network size only. Here synapses number are raised, and concomitantly, finite-size effects are reduced. We thus obtain effects combining those described in **B** and **H**. **(F)** Varying the synaptic weights of recurrent excitatory synapses. **(G)** Varying the synaptic weights of recurrent inhibitory synapses. **(H)** Varying network size (N_{tot}) and the connectivity probability simultaneously (with $N_{tot} \propto \frac{1}{p}$) so that the number of synapses per neuron is kept equal. In this setting, the average statistical quantities of populations activity (ν_e, ν_i and $|I_i/I_e|$) are kept identical, only finite size effects are reduced (see details in **Figure S1**). **(I)** Varying the strength of the afferent connectivity to the disinhibitory population (i.e. modulating the coupling between the afferent population and the disinhibitory population).

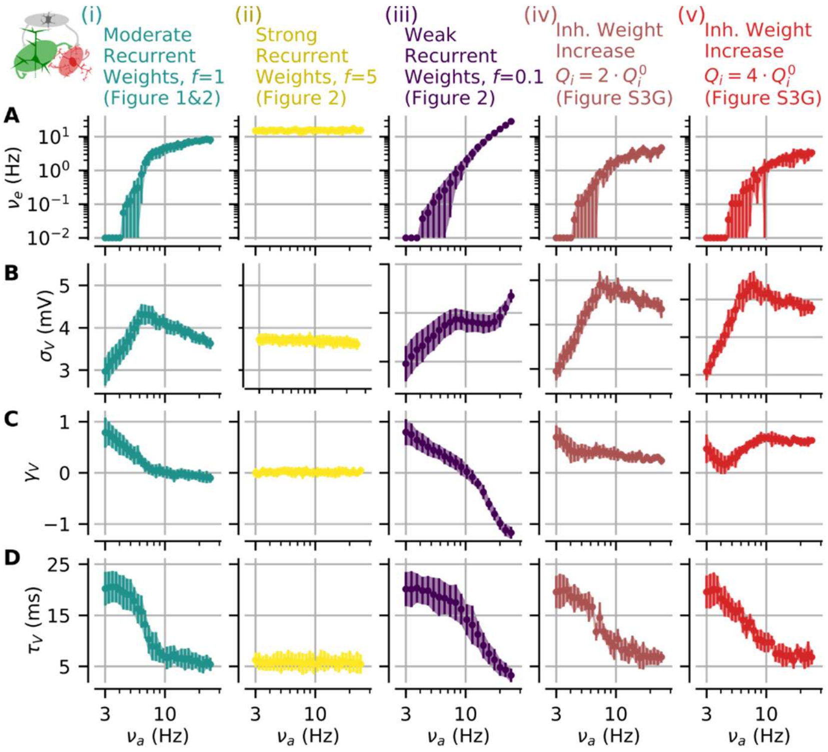

Figure S4. Relationship between membrane potential features and firing rates as function of the afferent rate for different parameter settings of the recurrent network. Related to Figure 3. From left to right, we analyzed network with different parameter settings: **(i)** moderate and balanced recurrent interactions (the reference architecture shown in **Figure 1, 2**); **(ii)** strong and balanced recurrent interactions found for f=5 in **Figure 2**; **(iii)** weak and balanced recurrent interactions found for f=0.1 in **Figure 2**; **(iv)** a moderately inhibitory-augmented case with Q_i=2· Q_{i0}; **(v)** a strongly inhibitory-augmented case with Q_i=4· Q_{i0}. From top to bottom: **(A)** relationship between afferent activity, ν_a, and the single cell firing rate, ν_e; **(B)** relationship between ν_a and the amplitude of the fluctuations, σ_V; **(C)** relationship between ν_a and the skewness of the V_m fluctuations, γ_V; **(D)** Relationship between ν_a and the autocorrelation time of the V_m fluctuations, τ_V. The mean ± s.e.m. over 10 excitatory cells averaged over 10 network simulations lasting 1s each is shown.

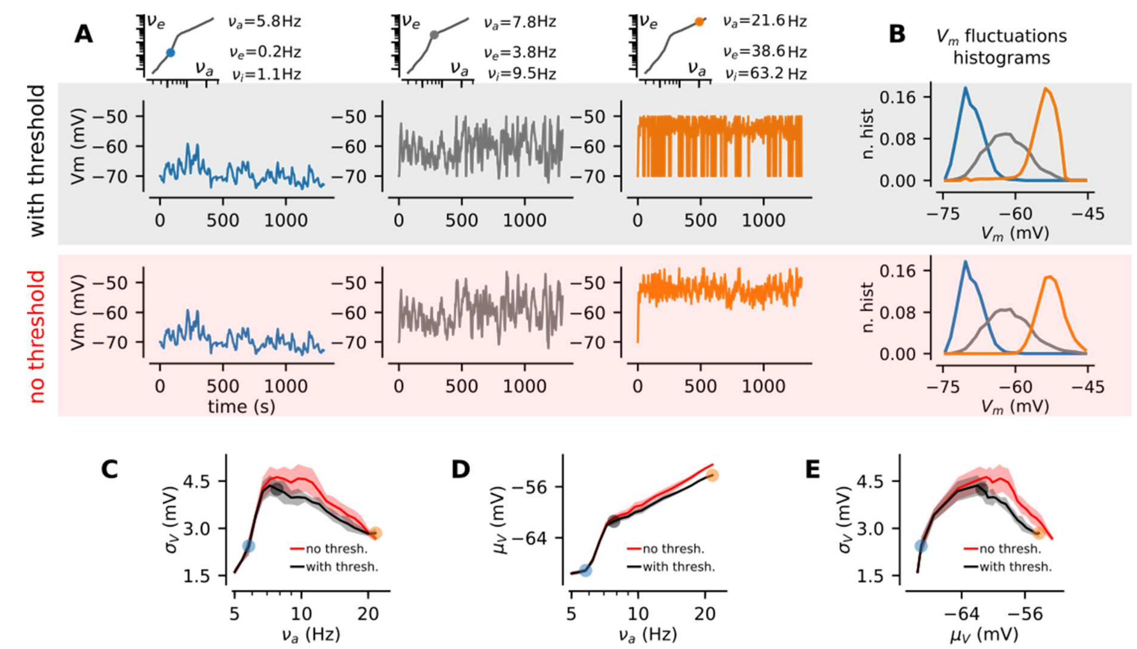

Figure S5. Effect of the threshold-and-reset mechanism on the relationship between μ_V and σ_V in the theoretical model. Related to Figure 4. (A) Simulation of single cell integration (Equation 1 in **Methods**) with (top) and without (bottom) the threshold-and-reset mechanism at three levels of afferent activity and recurrent activity along the spectrum (see annotations on top of the plot, the relationship between ν_a and ν_e or ν_i have been extracted from the simulations shown in **Figure 4B**). **(B)** Histogram with (top) and without (bottom) the threshold-and-reset mechanism at three levels of afferent activity (the color code for the different levels is as in **A**). Note that all histograms and their properties (i.e. μ_V and σ_V) reported in the manuscript were evaluated by blanking the refractory period following spikes to remove the impact of the artificial refractory mechanism of the integrate and fire model (see **Methods** and the resulting histograms in **Figure 4G**). **(C)** Estimated standard deviation σ_V as a function of the level of afferent input ν_a (associated to increasing recurrent excitation and inhibition, see **Figure 4B**) with (black) and without (red) the threshold-and-reset mechanism. Simulations lasted 10s and were performed with a time step of 0.01ms and repeated over 4 seeds for the Poisson processes. The mean $\pm$ s.e.m over those 4 seeds are provided. **(D)** Estimated mean depolarization level μ_V as a function of the level of afferent input ν_a with (black) and without (red) the threshold-and-reset mechanism. **(E)** Estimated relationship between μ_V and σ_V with (black) and without (red) the threshold-and-reset mechanism. In **C-E**, the colored dots represent the levels of afferent activity color-coded as the lines shown in **A** and **B**, which were derived at levels of afferent activity corresponding to the dots.

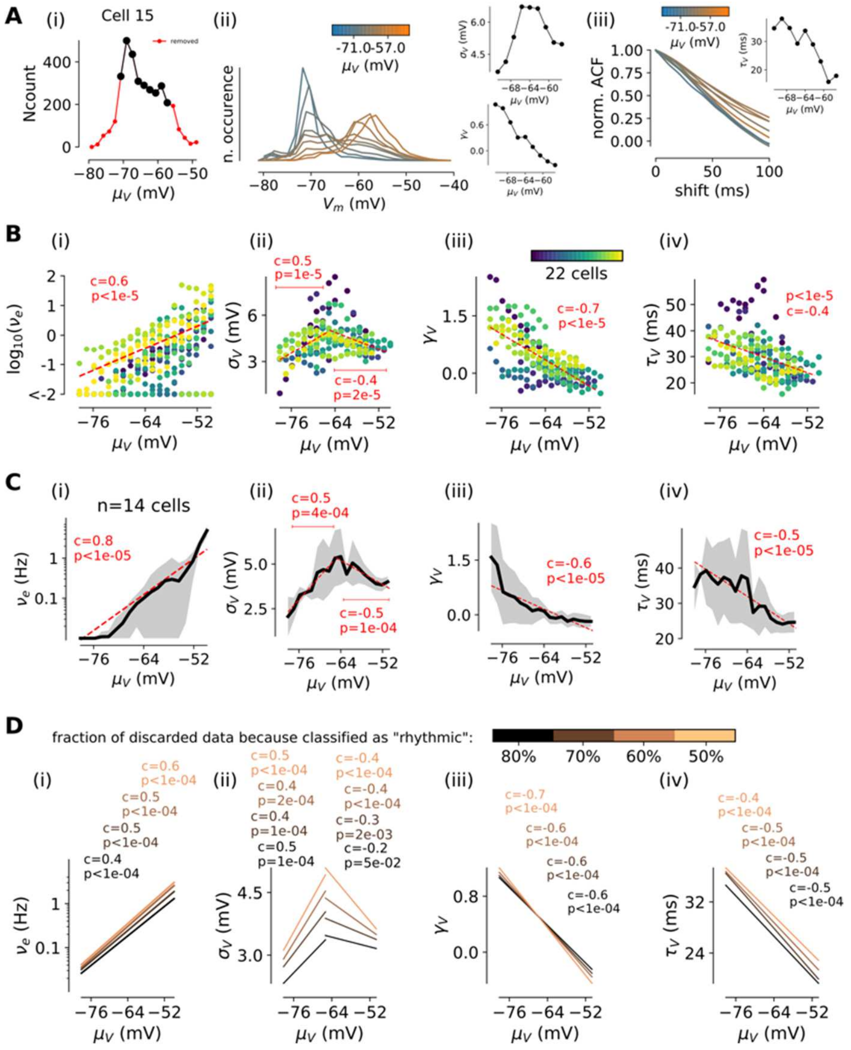

Figure S6. Characterization of the electrophysiological signature of non-rhythmic epochs in awake mice S1. Related to Figure 6. (A) Illustration of the data analysis on a given cell: “Cell 15”. (i) We count the number of non-rhythmic segments at different levels of mean depolarization μ_V. We keep the levels showing more than 200 segments (black) and discard the levels that have a too low occurrence to be analyzed (in red, N < 200, see Methods). (ii) After being classified as non-rhythmic and associated to a given μ_V level (color-coded), the V_m samples are included in the pooled distribution, that will determine σ_V and γ_V, see right insets. (iii) Concomitantly, the V_m autocorrelation function of all segments increments the pooled autocorrelation function (“norm. ACF” for normalized autocorrelation function) whose integral will determine τ_V (see details in the Methods). **(B)** Spiking activity (i) and V_m fluctuations signature (ii, iii, iv) for all single recordings data (n=22 cells). Note that, in (i), to perform the linear regression between μ_V and $\log_{10}(\nu_e)$, a 0.01 Hz baseline value was added to all single cell data to prevent 0 Hz values. **(C)** Ensemble data for the cells corresponding to Wild-Type mice only (n = 14 cells). Note that all correlations discussed in the main text are equally present in this reduced dataset ($|c| \geq 0.5$ and p < 1e-3 for all relations). **(D)** Varying the threshold for the classification of “non-rhythmic epochs”. We increase the threshold to discard up to 80% of the data. Note that all correlations discussed in the main text are equally present in this very conservative setting (black, fraction = 80%, p $\leq$ 5e-2 for all relations).

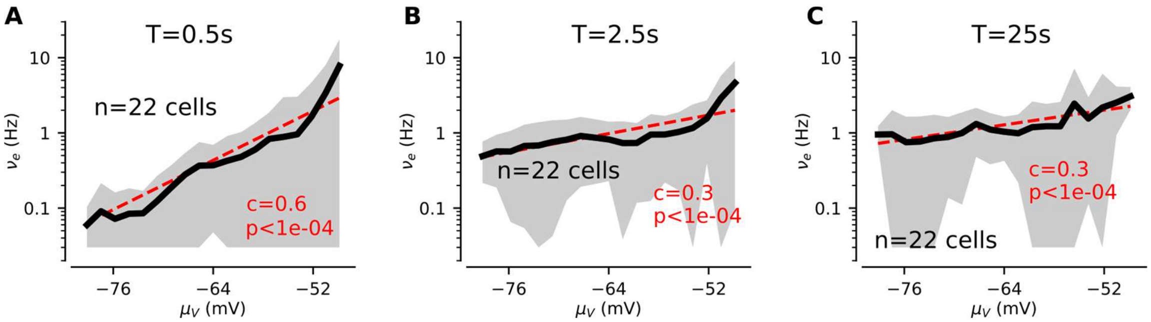

Figure S7. Effect of the extent of the sliding window (T) on the relation between depolarization level μ_V and firing rate ν_{out} in the analysis of non-rhythmic epochs. Related to Figure 6. We reproduce the analysis of Figure 6A (shown in panel **A,** T=0.5s) and we vary the window extent from T=0.5s to T=2.5s (panel **B**) and T=25s (panel **C**). Note how restricting the analysis to the slow component of the dynamics (visible for T=25s) produces a reduction of the range of observed firing rates per μ_V level. Similarly to **Figure 6A**, we performed least-square linear regressions (red dashed curves) on the log-transformed data and we reported the correlation coefficients of the linear regression ("c", see annotations in panels **A-C**). We evaluated statistical significance ("p") with a non-parametric one-tailed permutation test (performed with 1e4 permutations).

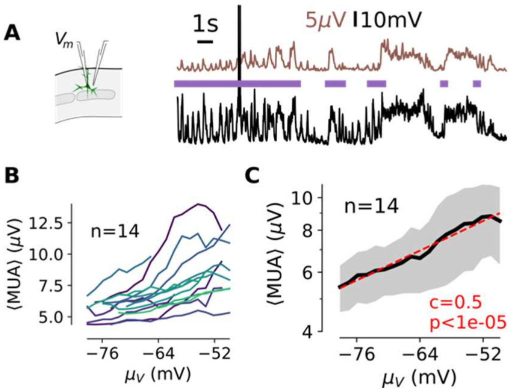

Figure S8. The average multi-unit activity ⟨MUA⟩ in layer 2-3 exhibits an exponential scaling with respect to the depolarization level μ_V across the non-rhythmic epochs of wakefulness in mouse barrel cortex. Related to Figure 6. (A) We show a representative recording of the simultaneous membrane potential (V_m, black) and multi-unit activity (MUA, brown) traces in layer 2-3. The MUA was extracted from the raw extracellular recording by bandpass filtering in the 300-3000 Hz band, then full-wave rectified, and smoothed with a 20 ms Gaussian filter. The purple line highlights the discarded rhythmic periods (see Methods). **(B)** Average relationships between the depolarization level μ_V and the mean MUA for all cells (color-coded, n = 14) . **(C)** Resulting population data (n = 14 cells). Note the logarithmic scale on the y-axis. The correlation coefficients and the p-value of a one-tailed permutation test (see **Methods**) are shown in the panel.

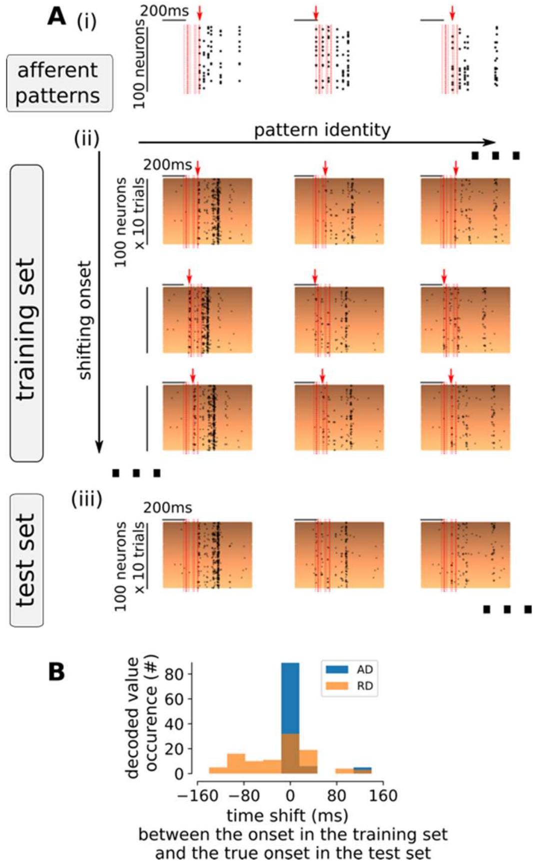

Figure S9. Decoding the spatio-temporal features of the afferent patterns: decoding pattern identity and onset timing. Illustrated on the AD regime. Related to Figure 7. (A) We show three different afferent patterns (i) together with their corresponding output patterns in the training set (ii) and test set (iii). One column corresponds to a given afferent pattern in ii-iii. The dashed red lines correspond to all onset times present in the set of afferent patterns. **(i)** Raster plots of the afferent patterns. We highlight the onset time of a given pattern with a red arrow. **(ii)** We show the 9 output spiking patterns in the training set corresponding to the 3 input patterns and the 3 onsets shown in (i) (raster activity represented both across the 100 neurons of the subpopulation and 10 trials, representation identical to **Figure 7B-C**, the color level identifies a given neuron and its activity over trials is duplicated along the y-axis). Note that the various arrows indicate the various onsets. **(iii)** We show the output patterns of the sub-network in the test set (100 neurons per 10 trials, representation as in (ii)). A correct decoding of the pattern identity corresponds to the association of the test pattern with a trial from the diagonal of the training set pattern (corresponding to the matching pattern identity and the matching stimulus onset). **(B)** Histogram of the error (with respect to the true onset) made in decoding times of onset of the afferent stimulus in that trial (called "time shift" on the x axis legend) over all trials in the test set. Note the very accurate decoding of the afferent stimulus onset in the AD regime (large peak at 0-shift, i.e. correct decoding), and the very inaccurate decoding of onset time in the RD regime.

Supplementary Tables

	Parameter	Value
Properties of excitatory cells	C_m	200 pF
	g_L	10 nS
	E_L	-70 mV
	V_{reset}	-70 mV
	V_{thre}	-50 mV
Properties of inhibitory cells	C_m	200 pF
	g_L	10 nS
	E_L	-70 mV
	V_{reset}	-70 mV
	V_{thre}	-53 mV
Connectivity probabilities	p_{ee}	5%
	p_{ei}	5%
	p_{ie}	5%
	p_{ii}	5%
	p_{ae}	10%
	p_{ai}	10%
Cell number	N_e	4000
	N_i	1000
	N_a	100
Synaptic parameters	Q_{ee}	2nS
	Q_{ei}	2nS
	Q_{ie}	10nS
	Q_{ii}	10nS
	Q_{ae}	4nS
	Q_{ai}	4nS
	E_e	0mV
	E_i	-80mV
	E_a	0mV
	τ_e	5ms
	τ_i	5ms
	τ_a	5ms

Table S1. Network parameters for the two population model (excitation/inhibition) with afferent excitation. Related to Figure 1,2,3.

	Parameter	Value
Properties of disinhibitory cell	C_m	200 pF
	g_L	10 nS
	E_L	-70 mV
	V_{reset}	-70 mV
	V_{thre}	-50 mV
Connectivity probability	p_{ad}	7.5%
	p_{di}	5%
Cell number	N_d	500
Synaptic parameters	Q_{ad}	4nS
	E_d	-80mV
	τ_d	5ms
	Q_{di}	10nS

Table S2. Additional parameters to construct the three population model (excitation/inhibition/disinhibition). Related to Figure 4,7.

(mV)	p_0	p_1	p_2	p_3	p_4	p_5	p_{11}	p_{12}	p_{13}	p_{14}	p_{15}	p_{21}	p_{22}	p_{23}	p_{24}
Excitatory cell	-51.5	2.5	-0.5	0.6	1.7	0.6	-2.8	-2.6	-0.1	-1.8	2.3	-2.6	-1.5	1.6	-0.1
Inhibitory cell	-54.0	1.9	-1.8	0.5	1.0	0.8	-2.3	-2.7	0.3	-1.5	1.3	-2.7	-1.1	1.3	-0.3

(mV)	p_{25}	p_{31}	p_{32}	p_{33}	p_{34}	p_{35}	p_{41}	p_{42}	p_{43}	p_{44}	p_{45}	p_{51}	p_{52}	p_{53}	p_{54}	p_{55}
Excitatory cell	1.8	-0.1	1.6	-1.3	0.6	0.1	-1.8	-0.1	0.6	0.0	-0.1	2.3	1.8	0.1	-0.1	-1.9
Inhibitory cell	1.8	0.3	1.3	-0.9	0.6	-0.1	-1.5	-0.3	0.6	0.0	0.3	1.3	1.8	-0.1	0.3	-0.8

Table S3. Fitted coefficients (in mV) entering in the input-output function (Equation 8 and 10) of the excitatory and inhibitory cells. Related to STAR Methods.
